# Umbilical Cord Blood Therapy Potentiated with Erythropoietin for Children with Cerebral Palsy: A Double-blind, Randomized, Placebo-Controlled Trial

**DOI:** 10.1002/stem.1304

**Published:** 2012-12-24

**Authors:** Kyunghoon Min, Junyoung Song, Jin Young Kang, Jooyeon Ko, Ju Seok Ryu, Myung Seo Kang, Su Jin Jang, Sang Heum Kim, Doyeun Oh, Moon Kyu Kim, Sung Soo Kim, MinYoung Kim

**Affiliations:** a Department of Rehabilitation Medicine, CHA UniversitySeongnam-si, Gyeonggi-do, Korea; b Department of Laboratory Medicine, CHA UniversitySeongnam-si, Gyeonggi-do, Korea; c CHA Medical Center Cord Blood BankGyeonggi-do, Korea; d Department of Nuclear Medicine, CHA UniversitySeongnam-si, Gyeonggi-do, Korea; e Department of Radiology, CHA UniversitySeongnam-si, Gyeonggi-do, Korea; f Department of Internal Medicine, Division of Hematology-Oncology, CHA UniversitySeongnam-si, Gyeonggi-do, Korea; g Department of Pediatrics, Division of Hematology-Oncology, CHA Bundang Medical Center, CHA UniversityGyeonggi-do, Korea; h Seoul CRO Co., Ltd.Seoul, Korea

**Keywords:** Umbilical cord blood, Erythropoietin, Cerebral palsy, Clinical trial, Function

## Abstract

Allogeneic umbilical cord blood (UCB) has therapeutic potential for cerebral palsy (CP). Concomitant administration of recombinant human erythropoietin (rhEPO) may boost the efficacy of UCB, as it has neurotrophic effects. The objectives of this study were to assess the safety and efficacy of allogeneic UCB potentiated with rhEPO in children with CP. Children with CP were randomly assigned to one of three parallel groups: the pUCB group, which received allogeneic UCB potentiated with rhEPO; the EPO group, which received rhEPO and placebo UCB; and the Control group, which received placebo UCB and placebo rhEPO. All participants received rehabilitation therapy. The main outcomes were changes in scores on the following measures during the 6 months treatment period: the gross motor performance measure (GMPM), gross motor function measure, and Bayley scales of infant development-II (BSID-II) Mental and Motor scales (18). F-fluorodeoxyglucose positron emission tomography (^18^F-FDG-PET/CT) and diffusion tensor images (DTI) were acquired at baseline and followed up to detect changes in the brain. In total, 96 subjects completed the study. Compared with the EPO (*n* = 33) and Control (*n* = 32) groups, the pUCB (*n* = 31) group had significantly higher scores on the GMPM and BSID-II Mental and Motor scales at 6 months. DTI revealed significant correlations between the GMPM increment and changes in fractional anisotropy in the pUCB group. ^18^F-FDG-PET/CT showed differential activation and deactivation patterns between the three groups. The incidence of serious adverse events did not differ between groups. In conclusion, UCB treatment ameliorated motor and cognitive dysfunction in children with CP undergoing active rehabilitation, accompanied by structural and metabolic changes in the brain. Stem Cells*2013;31:581–591*

## INTRODUCTION

Cerebral palsy (CP) is the leading cause of disability in early childhood. CP describes a group of disorders typically characterized by abnormal movement and posture attributed to nonprogressive disturbances that occur in the developing brain with associated disabilities including cognitive impairments [1, 2]. Despite extensive treatment, the neurological impairments eventually lead to lifelong functional deficits [3]. A recent report suggested that these impairments may be overcome by stem cell therapy [4].

Among stem cell sources, umbilical cord blood (UCB) reportedly contains stem cells with variable therapeutic potential [5]. Since its first use in Fanconi anemia in 1988 [6], UCB has been widely used as a source of hematopoietic stem cells. UCB has also been administered to children with metabolic disorders involving cerebral dysfunction [7]. The stem cells in UCB were also effective against various neurologic diseases [8–11]. An experimental study in an animal model of CP showed therapeutic effects of intraperitoneally administered UCB cells, with incorporation of these cells into the brain lesion [12]. Emerging clinical reports have also shown the feasibility of hematopoietic cell application, including UCB, for pediatric brain lesions [13–15].

UCB cells have been banked worldwide and no harmful effects have been reported [7]. In terms of therapeutic purposes for CP, UCB has potential based upon its known neuroprotective properties from anti-inflammatory and anti-apoptotic activities [16]. The immature brain responds to injuries with distinctively detrimental effects of inflammation and apoptosis [17]. And the pathologic response seems to persist several years; children with CP (mean ± SD age, 7.2 ± 3.6 years) had increased systemic inflammatory responses [18]. Thus, UCB might be effective in treating children with CP. While autologous UCB would be ideal for this purpose, most children with CP do not have banked UCB. Allogeneic UCB transplantation could be an alternative treatment option, but it is somewhat limited by immunological problems. To date, allogeneic UCB has been transplanted with the addition of a myeloablative chemotherapy regimen [19], which is associated with a high-risk of mortality [20]. Following reports showing functional improvements with only cyclosporine administration in animal experiments [8, 9], we concluded that myeloablative therapy may not be required for this purpose. In addition, we searched for the optimal candidate of adjuvant therapy that might potentiate the stem cell action of UCB.

For neural recovery, combination therapy with neurotrophic factors may potentiate cell therapy efficacy [21–23]. Erythropoietin (EPO), derivatives of EPO, and granulocyte-colony stimulating factors could potentially be delivered in a clinical trial [13, 24, 25]. Among these factors, we chose EPO as an adjunct to UCB therapy because EPO has neuroprotective and neural-repair properties, particularly in neonatal hypoxic/ischemic brain injury and in CP models [26–29]. EPO stimulates Jak2-PI3K-Akt [30] to exert its neuroprotective action, which is similar to the pathway stimulated by UCB stem cells [31].

Thus, this trial was conducted to determine the efficacy of UCB treatment with concomitant adjunctive rhEPO administration in children with CP. While this trial was intended to test the efficacy of UCB with possible enhancement by rhEPO, the independent effects of rhEPO and placebo were also assessed. Any possible adverse events caused by these treatments were monitored. Ongoing rehabilitation was also considered important for the neurodevelopmental and functional improvement of the children in this study.

## MATERIALS AND METHODS

### Participants

The inclusion criteria were a diagnosis of CP according to clinical history and physical examination, age between 10 months and 10 years (mean ± SD: 39.8 ± 20.9; median: 36.5 months), and written informed consent from parents. The exclusion criteria were pneumonia or renal dysfunction at enrollment, any known genetic disorder, known allergy to any of the study medications, clinically obvious intractable epilepsy, lack of adequate family support (including the ability to attend follow-up visits), and any other features that hampered the interpretation of results according to the clinical judgment of the principle investigator (PI). This trial was registered at http://www.clinicaltrials.gov (NCT01193660).

### Study Design and Masking

The procedure was designed and conducted as a placebo-controlled, double-blind study. This study was approved by the institutional review board of the CHA Bundang Medical Center, Korea. Between May 30 and November 30, 2010, 105 children with CP were enrolled, hospitalized, and randomly assigned to one of three parallel treatment groups of equal size. The sample size was determined without considering statistical significance due to the limited number of volunteers. For even distribution among the three groups, the children were preferentially divided into 22 units according to age, function, and spasticity. The children were randomly distributed (i.e., 1:1:1 allocation) by an independent provider for each unit who was not informed about each subject. The first group received UCB potentiated with rhEPO (Espogen®, LG life sciences, Seoul, Korea of Republic) and rehabilitation and was referred to as the pUCB group. The second group received rhEPO, placebo UCB, and rehabilitation and was referred to as the EPO group. The third group received rehabilitation only with placebo UCB and placebo rhEPO and was referred to as the Control group.

In accordance with a placebo-controlled double-blind trial protocol, all participants including family members, observers, investigators, and employees were blinded to the group assignment. The only exceptions were the attending physician and the charge nurses who provided the patients with the medications necessary to maintain their health, who were independent of this trial. They did not provide any information concerning the group assignment to anyone. This masking design was adopted because the subjects were fragile children who were medically at risk. The computer system in the hospital was shielded against exposure of the groupings. Within a week after the baseline evaluation, the true or placebo UCB and rhEPO therapies commenced. As placebo materials, autologous peripheral blood was used for UCB, and normal saline was used for rhEPO. Albumin (5%, 0.5 ml in 150 ml saline) was used as placebo for intravenous cyclosporine. An oral solution of cyclosporine was given with orange juice, and placebo for oral cyclosporine was juice alone. Placebo materials could not be differentiated by their appearances.

The initial evaluation of the participants consisted of various functional CP measurements to establish a baseline (0 month). The evaluations were repeated at 1, 3, and 6 months after the commencement of treatment. At baseline, each participant underwent brain diffusion tensor imaging (DTI) and ^18^F-fluorodeoxyglucose positron emission tomography (^18^F-FDG-PET/CT). ^18^F-FDG-PET/CT and DTI were repeated to determine changes in the brain at 2 weeks and 6 months post-treatment, respectively. All participants received an intensive 1-month in-patient rehabilitation program consisting of two sessions of physical and occupational therapy per day. After discharge, each participant continued to receive rehabilitation therapy at least 3 days per week until the last follow-up assessment at 6 months post-treatment.

### UCB Infusion and EPO Therapy

Unrelated allogeneic UCB units were selected from the affiliated UCB bank, the CHA Medical Center Cord Blood Bank. The UCB units consisted of at least 3 × 10^7^/kg total nucleated cells (TNCs), matched for at least four of six human leukocyte antigen (HLA) types A, B, and DRB1. Before UCB administration, each unit was washed to eliminate dimethyl sulfoxide [32]. A single intravenous infusion of true or placebo UCB was performed for each subject by the PI. The pUCB group received cyclosporine intravenously at 3 mg/kg per day for 6 hours twice a day during the first week, commencing 12 hours before the UCB infusion, and continued oral cyclosporine solution (12 mg/kg per day) for the following 3 weeks.

All participants in the pUCB and the EPO groups received two rhEPO injections at a dose of 500 IU/kg at 2 and 12 hours before the UCB or placebo infusion. Subsequently, from day 3 on, each received rhEPO subcutaneously twice per week for 4 weeks in 250 IU/kg doses. If the hemoglobin level was elevated above 15 g/dl on weekly complete blood cell count monitoring, the rhEPO was stopped. All participants were followed up for at least 1-year after their enrollment to monitor any adverse events.

### Functional Assessments

Four main assessments included total scores on the gross motor performance measure (GMPM) [33], gross motor functional measure (GMFM) [34], and raw scores on Mental and Motor scales of the Bayley Scales of Infant Development-II (BSID-II) [35]. The GMPM and GMFM were used to measure gross motor ability, and the BSID-II was used to measure neurodevelopmental progress. Reliability tests for the main outcome measures were performed before the intervention. The GMPM inter-rater reliability intraclass correlation coefficients (ICCs) were 0.85–0.92 (*n* = 75, 10 raters), and the inter-rater reliability ICCs for the BSID-II Mental and Motor scale scores were 0.92–0.99 (*n* = 68, 10 raters). The inter-rater reliability ICCs of the GMFM subscores and total score were 0.97–1.00 (*n* = 101, 10 raters) and the intrarater reliability ICCs were 0.99–1.00 (*n* = 101, two raters). The main outcomes were changes in these scores from the baseline to the final assessment.

Typical movements at each assessment were recorded using a digital camcorder. The participants were also assessed using the following: the pediatric evaluation of disability inventory (PEDI) [36], the functional independence measure for children (WeeFIM) [37], the sumed scores on the manual muscle strength test (Supporting Information Contents 2), and the quality of upper extremity skills test [38].

### DTI and Fractional Anisotropy

All available participants were scanned using a 3T GE Signa System (General Electric, Milwaukee, WI) for routine magnetic resonance imaging (MRI), and a neuroradiologist reported the findings. DTI data were also acquired using two-dimensional axial spin-echo planar imaging with refocusing pulses. The sequence parameters were repetition time/echo time of 12,000/108 ms; 1 number of excitations, 48 slices; 24-cm field of view; 128 × 128 matrix; 3.0 mm slice thickness; 25 gradient directions; *B* = 900; and a nondiffusion-weighted baseline image (*B* = 0). The imaging data were then processed using DTI studio software (Johns Hopkins University and Kennedy Krieger Institute, Baltimore, MD; http://www.mri.kennedykrieger.org).

Fractional anisotropy (FA) was measured by one physician at six different regions of interest: for the corticospinal tract, two loci in each posterior limb of the internal capsule (PLIC) were assessed by dividing them into anterior and posterior portions at the level of the globus pallidus, bilaterally (Supporting Information Contents 2); for the spinothalamic tract, the posterior lower pons loci were assessed, also bilaterally [39, 40]. Pretrial test-retest ICCs for the loci were 0.91–0.99 (*n* = 50). The changes in FA from baseline to the 6-month measurement were used to determine the effects of the treatment on white matter integration.

### ^18^F-FDG-PET/CT Image Acquisition and Data Analysis

^18^F-FDG-PET/CT images were acquired using a Gemini PET/CT scanner (Phillips Medical Systems, DA Best, The Netherlands). Before radiotracer injection, a transmission scan was performed using CT to generate the attenuation maps. Approximately 50 minutes after the intravenous administration of 370 MBq of ^18^F-FDG and 30 minutes after sleep induction with chloral hydrate, 90 slices of brain emission images were obtained over a period of 20 minutes. The data from the PET images were analyzed by image reconstruction using the row action maximum likelihood algorithm. A board-certified nuclear physician reviewed the ^18^F-FDG-PET/CT scans. The spatial, preprocessing, and statistical analyses were performed using SPM8 implanted in Matlab R2011a (Mathworks, MA) to assess differences in the regional brain glucose metabolism between the groups and between the pretreatment and post-treatment image data. The ^18^F-FDG-PET images were converted from DICOM to the ANALYZE format using MRIcro (http://www.mricro.com) and transferred to SPM8 (Institute of Neurology, University College of London, U.K.). The data were then normalized to a standard PET template provided by SPM8. The standardized data were then smoothed using a Gaussian kernel (full-width half-maximum, 16 mm). The intergroup analyses were performed at baseline with a one-sample *t* test to check for differences between the groups. The changes in the images from pretreatment to post-treatment in each group were compared using paired *t* tests. Voxels with an uncorrected *p* < .05 were considered significant, and the extent threshold *K*_e_ was set at 100 voxels.

### Statistical Analyses

Statistical analyses were performed using SPSS version 19.0, (IBM, Chicago, IL, http://www.spss.com) and Prism 5.0, (GraphPad Software, San Diego, CA, http://www.graphpad.com). Depending on the normality of data, analysis of variance (ANOVA) or the Kruskal-Wallis and Mann-Whitney *U* tests were used to compare outcomes between the independent groups, and a paired *t* test was used to analyze intragroup differences. The categorical variables were analyzed using Chi-squared or Fisher's exact test. To evaluate correlations, Pearson's correlation analysis was performed.

## RESULTS

**Figure 1:**
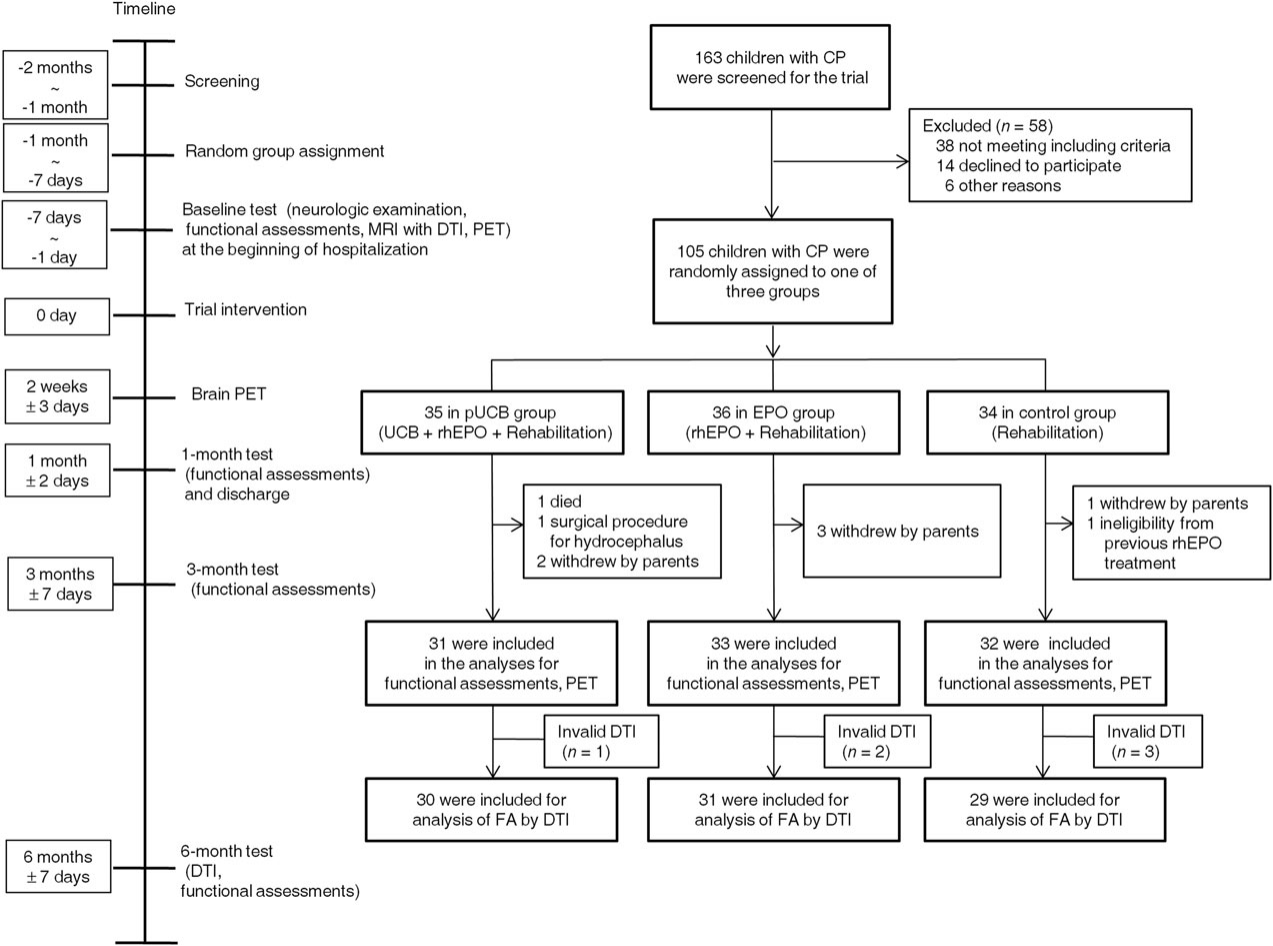
Study flow diagram. Abbreviations: CP, cerebral palsy; DTI, diffusion tensor image; EPO, erythropoietin; FA, fractional anisotropy; pUCB, UCB potentiated by rhEPO; PET, ^18^F-FDG PET (^18^F-fluorodeoxyglucose positron emission tomography); rhEPO, recombinant human erythropoietin.

Among the 105 children enrolled in this study, nine dropped out (Supporting Information Contents 1). Thus, 96 participants were included in the analyses (Fig. 1). There were no significant differences between the three groups, pUCB (*n* = 31), EPO (*n* = 33), and Control (*n* = 32), in the demographic data, MRI findings [41], severity of disease [42], typology [1], residence area, and duration of previous and postdischarge rehabilitation (Table 1; Supporting Information Table S1). The UCB, cyclosporine, and rhEPO were administered, as intended (Supporting Information Figs. S1, S2; Supporting Information Table S2). However, 20 of 71 rhEPO-treated participants discontinued rhEPO before the scheduled completion date due to elevated hemoglobin levels; these participants received a minimum of six injections.

**Table 1:** Demographic and baseline participant characteristics (*n* = 96)

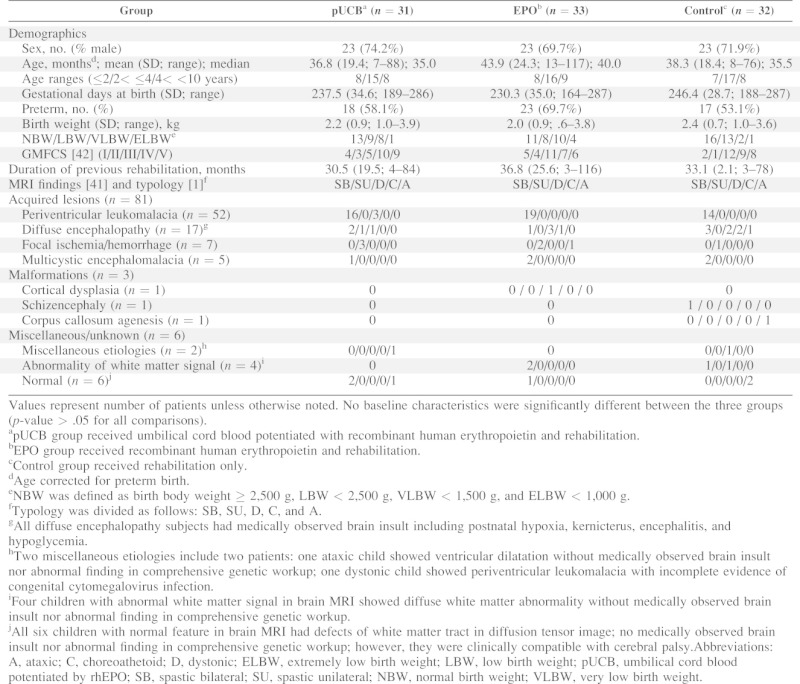

### Adverse Events

Ten serious adverse events that required the hospitalization of nine patients were reported among the 105 recruited participants; the distribution of these events did not differ between the three groups (Table 2). The death of a 25-month-old female patient occurred in the pUCB group at 14 weeks post-treatment. She was quadriplegic with spasticity from profound hypoxia with involvement of the central gray matter and brainstem, as shown by MRI (Supporting Information Contents 1). She had severe motor impairment and was unable to control her head. Due to poor oral motor function, tube feeding was required; however, her parents insisted on oral feeding, and obstructive phlegm was constantly present. She was medically stable post-treatment with continuous neurological improvement up until the 3-month follow-up evaluation. When she visited the pediatric neurology department for the routine seizure follow-up on the day of her death, she was found to be neurologically stable. Her death occurred during sleep with no apparent cause, and it was determined not to be related to the given treatment after scrutinizing all related records and events.

**Table 2:** Adverse events during the 6 months study period in the three groups (*n* = 105)

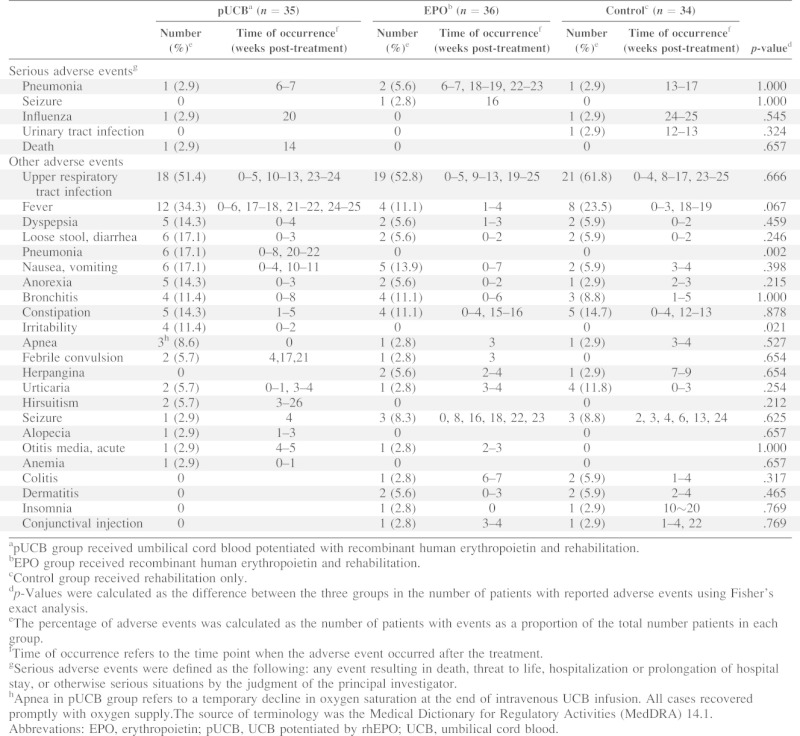

Regarding nonserious adverse events, pneumonia and irritability were more frequent in the pUCB group (Table 2). Hemoglobin levels were elevated in the rhEPO-treated groups without any thrombotic events (Supporting Information Fig. S2). General laboratory results did not indicate any adverse effects of UCB, cyclosporine, or rhEPO administration. At the 1-year follow-up evaluation, no prolonged or delayed onset of serious adverse effects was reported (Supporting Information Tables S3, S4).

### Efficacy of UCB and EPO Combination Therapy

There were no significant differences in baseline measurements between the three groups, including functional assessment scores, FA, and intergroup brain PET comparison results (Supporting Information Tables S5, S6; Supporting Information Fig. S3).

### Functional Improvement

**Figure 2:**
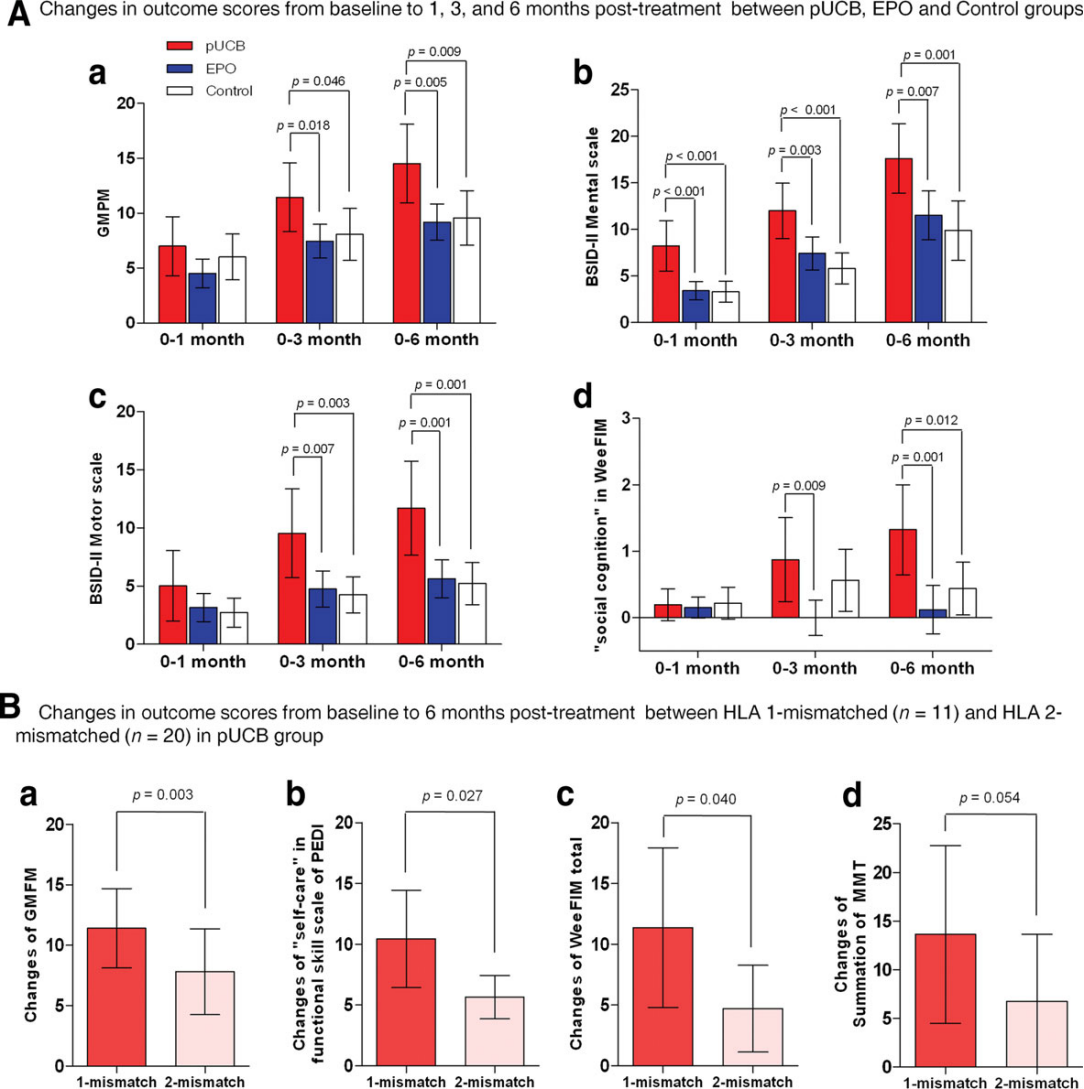
Comparing changes in outcome scores. Panel A shows changes in outcome scores from baseline to 1, 3, and 6 months post-treatment between pUCB, EPO, and Control groups. **(a):** GMPM total score, **(b)** BSID-II Mental scale raw score, **(c)** BSID-II Motor scale raw score, and **(d)** social cognition scale score of the WeeFIM. The pUCB group (*n* = 31) received umbilical cord blood potentiated with recombinant human erythropoietin and rehabilitation; the EPO group (*n* = 33) received rhEPO and rehabilitation; the Control group (*n* = 32) received rehabilitation only. Bars represent the 95% CI. The *p*-values compared changes in outcome scores between two groups based on post hoc analyses following ANOVA. Panel B shows changes in outcome scores for the period between baseline and 6 months post-treatment according to HLA mismatching in the pUCB group (*n* = 31). HLA 1-mismatched (*n* = 11) and HLA 2-mismatched (*n* = 20). **(a):** GMFM total score, **(b)** “self-care” score in functional skill scale of the PEDI, **(c)** WeeFIM total score, and **(d)** summation of MMT scores. Bars represent 95% CI. The *p*-values compare changes in outcome scores based on Mann-Whitney *U* test. GMPM scores range from 0 to 100, with higher scores indicating better motor performance. BSID-II scores range from 0 to 112 for Motor scale raw score and from 0 to 178 for Mental scale raw score, with higher scores indicating better motor and mental function, respectively. GMFM scores range from 0 to 100, with higher scores indicating better motor function. Scores on the social cognition scale of the WeeFIM range from 3 to 21, with higher scores indicating better social cognition. Total WeeFIM scores range from 18 to 126, with higher scores indicating greater functional independence. Self-care scores in functional skill scale of the PEDI range from 0 to 100, with higher scores indicating better function in self-care items. MMT summation scores range from 0 to 160, with higher scores indicating better muscle strength. Abbreviations: BSID-II, Korean version of the Bayley scales of infant development, second edition; GMFM, gross motor function measure; GMPM, gross motor performance measure; HLA, human leukocyte antigen; MMT, manual muscle strength test; PEDI, pediatric evaluation of disability inventory; rhEPO, recombinant human erythropoietin; WeeFIM, functional independence measure for children.

All three groups showed significant improvements in most of all functional measures over time (Supporting Information Table S6). The improvements over 6 months on the GMPM, the BSID-II Mental and Motor scales, and the “social cognition” scale in WeeFIM differed significantly among the groups (*p* < .009; Table 3). Multiple comparison tests for each difference revealed greater improvements in the pUCB group than in the EPO and Control groups (*p* < .010 for GMPM; *p* < .008 for BSID-II Mental scale; *p* < .002 for BSID-II Motor scale; *p* < .013 for social cognition of WeeFIM; Fig. 2A). Among the parameters that showed greater pUCB group improvements, the BSID-II Mental scale revealed differences starting at 1-month post-treatment (*p* < .001). Other measures showed differences beginning from 3 months post-treatment (*p* < .041; Table 3). Again, post hoc analysis revealed greater improvements in the pUCB group than in the EPO and/or Control groups (*p* < .047; Fig. 2A). Analysis of the changes in scores revealed differences in the GMFM score between the groups at the 3–6-month interval (*p* = .035) and post hoc analysis revealed greater improvement in the pUCB group than in the Control group (*p* < .05).

**Table 3:** Comparison of score differences in four main functional assessments and one subscale in the WeeFIM for the durations between each assessment period in three groups

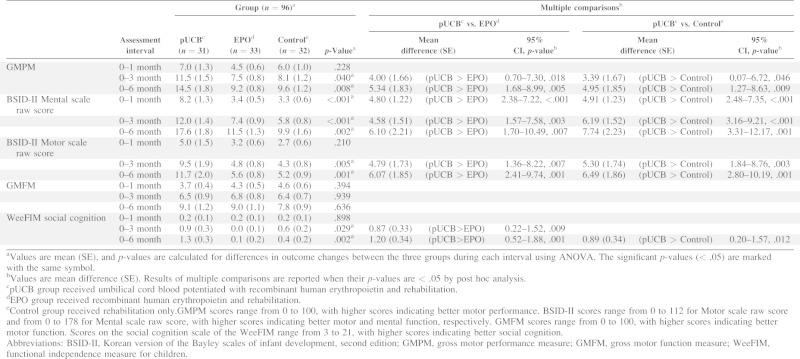

In-depth analyses were performed to examine differences in therapeutic responses according to demographic characteristics and the time intervals. Regarding age, when the children were divided into two groups by age, with the split at 36 months, the younger children showed greater improvements in the pUCB group than did the others on all main parameters for multiple intervals, while the older children showed improvements only in the BSID-II Mental scale during the 0–3-month period. Younger children, not only in the pUCB but also in the EPO group, showed better outcomes than the Control group on the GMFM at the 3–6-month interval (Supporting Information Table S7). When the participants were divided into two groups by birth history (i.e., preterm or full-term birth), the preterm group had better outcomes on the GMPM in the pUCB group during most intervals, and the full-term group had better outcomes on the BSID-II Mental scale in the pUCB group (Supporting Information Table S8). The more severely impaired group showed better outcomes on the BSID-II Mental scale in the pUCB group, whereas the less impaired showed better outcomes on the GMPM and BSID-II Motor scale in the pUCB group (Supporting Information Table S9). According to brain MRI findings of the presence or absence of an acquired lesion, no remarkable differences were observed in terms of superiority of the pUCB group (Supporting Information Table S10). When the patients were divided into two groups, as periventricular leukomalacia (PVL) and non-PVL group, the PVL group showed better outcomes in the pUCB group than in the EPO group on the GMPM, while the non-PVL group had better outcomes in the pUCB group on the BSID-II Mental scale (Supporting Information Table S11).

The influence of HLA incompatibility was also investigated by comparing HLA 1- and HLA 2-mismatched cases over the 0–6-month period. The HLA 1-mismatched group showed significantly better outcomes on the GMFM, the “self-care” score in functional skills scales of PEDI, and the WeeFIM total score (Fig. 2B). Higher TNC led to better outcomes on the GMFM, and more CD34+ cells led to better outcomes on the BSID-II Mental function from 1-month post-treatment and subsequently (Supporting Information Table S12).

### Structural Changes in DTI

**Figure 3:**
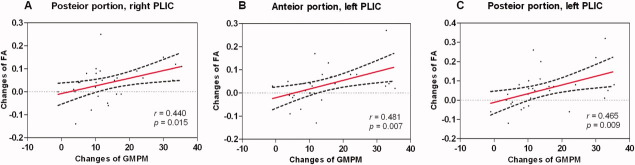
Correlation between changes of GMPM total score and changes of FA during the interval between baseline and 6 months post-treatment in pUCB (UCB potentiated by rhEPO) group (*n* = 30). The pUCB group received umbilical cord blood potentiated with recombinant human erythropoietin and rehabilitation. For FA, PLIC at three loci were measured. **(A):** Posterior portion, right side 95% CI of Pearson *r* = 0.094–0.691. **(B):** Anterior portion, left side 95% CI of Pearson *r* = 0.147–0.717. **(C):** Posterior portion, left side 95% CI of Pearson *r* = 0.125–0.707. Abbreviations: FA, fractional anisotropy; GMPM, gross motor performance measure; PLIC, posterior limb of the internal capsule.

The intragroup analysis showed significant FA increments in the pUCB group at all measured loci, but not in the other groups (*p* < .05; Supporting Information Table S5). The changes in the GMPM score at the 0–6-month interval were significantly correlated with the FA changes in the right posterior (*r* = 0.44, *p* = .015), left anterior (*r* = 0.48, *p* = .007), and left posterior (*r* = 0.47, *p* = .009) portions of the PLIC (Fig. 3). The one-way ANOVA revealed significant differences between the three groups with respect to changes in FA of the spinothalamic tract in the right posterior lower pons (*p* = .015), with the pUCB group showing greater increments than did the other groups (Supporting Information Fig. S4).

### Metabolic Changes in ^18^F-FDG-PET

**Figure 4:**
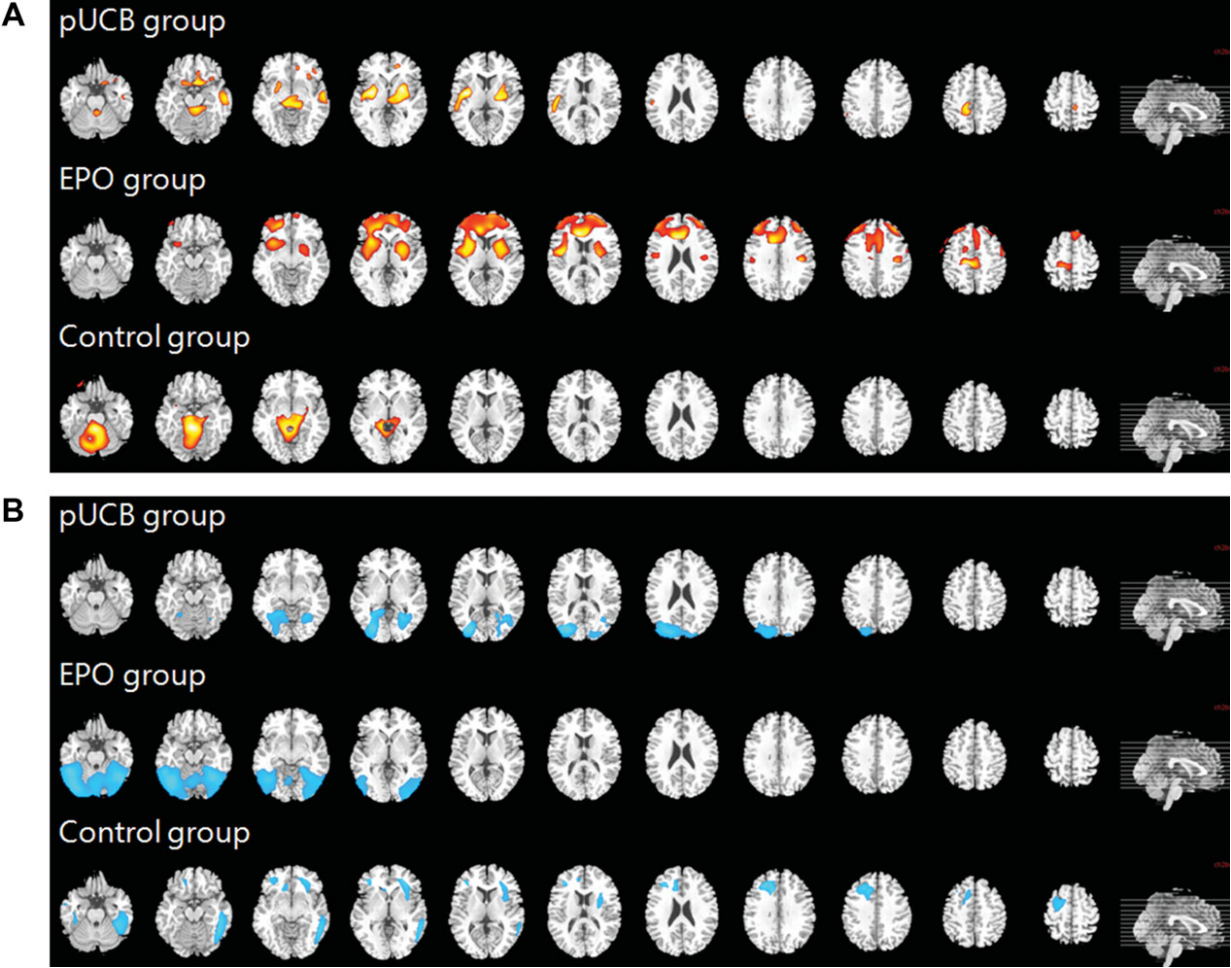
Changes in ^18^F-fluorodeoxyglucose positron emission tomography glucose metabolism during the period between baseline and 2 weeks post-treatment. The pUCB group (*n* = 31) received umbilical cord blood potentiated with recombinant human erythropoietin and rehabilitation; the EPO group (*n* = 33) received recombinant human erythropoietin and rehabilitation; the Control group (*n* = 32) received rehabilitation only. The template brain image was provided by SPM8.0. **(A):** Areas of increased glucose metabolism in the three groups (*p*-value < .05). Red and yellow denote areas with increased glucose metabolism. The pUCB group exhibited increased activity in the bilateral basal ganglia, thalami, and small areas in the bilateral frontal, right parietal, and left temporal lobes, whereas the EPO group showed increases in a large area in the bilateral frontal lobes and basal ganglia, and the Control group showed increases only in the bilateral cerebelli. **(B):** Areas of decreased glucose metabolism in the three groups (*p*-value < .05). Blue denotes areas with decreased glucose metabolism. The pUCB group exhibited decreased activity in the right occipital lobe and the bilateral parahippocampal gyri, whereas the EPO group showed decreased activity in the bilateral cerebelli, and the Control group showed decreased activity in the bilateral frontal and temporal lobes. Abbreviations: EPO, erythropoietin; pUCB, UCB potentiated by rhEPO.

All participants underwent ^18^F-FDG-PET. Different areas of increased and decreased activity were observed in each of the three groups. Noticeable increases were observed in the basal ganglia and the thalamus in the pUCB group. The EPO group showed increased activity in a large area of the bilateral frontal lobes, and the Control group showed increased metabolism in the cerebellum only. The areas of decreased activity included the occipital and limbic lobes in the pUCB group, the occipital lobes in the EPO group, and multiple areas of the frontal and temporal lobes in the Control group (Fig. 4; Supporting Information Table S13).

## DISCUSSION

In this study, allogeneic UCB infusion potentiated with rhEPO ameliorated motor and cognitive impairment in children with CP, suggesting that this strategy could be developed as a novel therapeutic approach. A comprehensive evaluation of the adverse effects of this therapy is, however, necessary before its clinical application. Higher rates of nonserious adverse events (i.e., pneumonia and irritability) were reported in the pUCB group. As this is concordant with previously reported adverse effects of cyclosporine [43], it seems that the occurrences resulted from cyclosporine treatment rather than from UCB. The transient declines in oxygen saturation in the pUCB group were observed just after intravenous UCB administration and were thought to be related to first-pass lung sequestration, although the incidences did not differ between the three groups. All these events were promptly resolved by providing oxygen supply. No differential incidence with serious adverse events was observed. One death among the participants occurred in the pUCB group. The patient had severe neonatal hypoxic ischemic encephalopathy (HIE). She had severely impaired oropharyngeal function with frequent episodes of aspiration; therefore, suffocation was the most likely cause of death due to saliva or regurgitated food. Her profound HIE condition per se could confer high-risk for death, as the death rate is approximately 50% in neonatal HIE with deep nuclear involvement within 3 years of age. Additionally, the risk is increased by brainstem involvement in these cases [44], and this single mortality had the mark of HIE in the brain stem in MRI. The patient did not show any signs of functional decline, seizure aggravation, or any laboratory abnormalities (Supporting Information Contents 1). Considering the overall frequency and severity of the adverse effects in this study, the risks did not appear to be prohibitive in considering this new approach for CP.

In terms of therapeutic efficacy, the data consistently reveal superior outcomes in the pUCB group compared with both the EPO and Control groups; these differences were significant starting from 1-month or 3 months post-treatment and continued to 6 months post-treatment. The beneficial effects appeared not only in motor function but also in cognitive function. Regarding motor outcome, the GMPM and BSID-II Motor scores showed remarkable changes from 3 months post-treatment, and improved GMFM was noticeable during the 3–6 months period in the pUCB group. Regarding cognitive outcome, the pUCB group showed improvements on the BSID-II Mental scale as early as 1-month post-treatment; however, the effect on daily living appeared at 3 months post-treatment with an elevated social cognition score on the WeeFIM. The EPO group had no superior outcomes in the overall comparisons, except for GMFM during the 3–6-month period in young children less than 36 months old, suggesting that the efficacy observed in the pUCB group was derived predominantly from UCB. The effect of rhEPO was apparently minimal, although it was expected to exert therapeutic efficacy, and the administered dosage or age factor could have been related to the result [45, 46].

Our in-depth analyses suggested that the therapeutic effects of this novel approach act differentially on the brain under specific conditions. Children younger than 36 months of age seemed to benefit more from UCB and EPO treatment than did the older children, as seen in their more favorable responses. Patients with preterm-related CP had better motor outcomes, whereas full-term patients with CP had better cognitive outcomes. CP patients with less severely impaired gross motor function benefited more in motor function, whereas the more severely physically impaired children benefited more in mental function. PVL lesions, the most representative cause of CP, were associated with higher GMPM scores. However, when the group was divided according to brain MRI findings into acquired and nonacquired lesions, no differential outcomes were observed.

One interesting finding was the influence of HLA incompatibility, although the numbers of subjects in the two subgroups were small. As many outcome scales present favorable responses when HLAs are matched, immunity is a considerable factor in allogeneic cell therapy. This finding also suggests great potential for autologous UCB treatment in patients with CP [13, 14]. CD34+ and total cell number also affected outcomes; greater cell number led to a better response. However, the optimal cell number could not be determined by this study.

As for the therapeutic mechanism of this novel approach, intravenously infused cells may act as remote “bioreactors” that eventually increased systemic anti-inflammatory cytokine production after being sequestrated in the lung microvasculature and parenchyma and entrapped in the spleen via direct stimulation of pulmonary macrophage and spleen-naive T cells [47]. This study did not focus on the direct mechanism of the stem cell therapy effectiveness. Instead, we suggest indirect evidence through the use of brain-imaging data. Given that the FA increment in the sensory and corticospinal tract analyses was correlated with increments in the GMPM score, increased axon density or enhanced myelination may be a possible explanation. FA indicates white matter integrity, and the structural integrity of the corticospinal tract is correlated with the clinical severity of CP [48]. With respect to metabolic changes during the first 2 weeks post-treatment, the ^18^F-FDG-PET analyses revealed distinguishable patterns between the groups. Wide enhancement in the frontal lobes of the EPO group might have been due to dopaminergic stimulation [49, 50], whereas the cerebellar activation in the Control group could have resulted from intensive exercise [51]. The pUCB group showed enhanced metabolism in the basal ganglia and the thalamus; this may be meaningful because a previous SPECT study showed decreased perfusion in the same areas [52]. Considering that rhEPO was administered to both the pUCB and EPO groups, the different findings between the two groups could have caused by UCB action that affected complex brain network.

There are potential limitations of this study. Importantly, a UCB-alone group, which would have allowed an estimate of the respective contributions of UCB and rhEPO, was not included. The purpose of the study was to observe maximized UCB efficacy in fragile children with CP in a double-blind setting, while the number of participants was limited. Practically, we were only capable of allocating up to three groups. Further research demonstrating the efficacy of UCB alone is needed. The distribution of subject characteristics, including age, pathological causes of CP, severity, typology, and MRI findings was wide, although the parameters were matched among the three groups, and response differences according to the characteristics were analyzed in depth. Issues regarding cyclosporine use may also be raised. To date, no research has indicated an appropriate duration of immunosuppression for cell therapy. A previous study of systemic UCB administration for brain injury revealed no remaining cells 1-month after the therapy, even with continuous cyclosporine administration [53]; thus, immunosuppression for longer than a month seemed unnecessary. Cyclosporine may cause many adverse effects, although in this study they were not serious. Conversely, some reports showed neuroprotective effects of cyclosporine and activation of the AKT pathway, similar to EPO [30, 54, 55], which showed stronger neuroprotection in combination [56]. Thus, this mechanism could have also contributed to the UCB efficacy in the pUCB group, even though extended long-term use of cyclosporine solely for this purpose cannot be recommended due to its eventual neurotoxicity [57]. Further studies should be performed to delineate long-term effects of UCB, to examine differences in efficacy between UCB of autologous and allogeneic origins, and to elucidate the mechanism of UCB.

## CONCLUSIONS

This trial assessed the effect of allogeneic UCB therapy for children with CP, and the results suggested potential benefits of this new approach. Improvements in cognitive and motor function were witnessed in CP patients without significant harmful events.

## Supplementary material

Supporting Information Figure 1. Cyclosporine levels during four weeks of immunosuppression in pUCB group Horizontal bars and shaded area denote median values and the target range of level control (100 to 200 ng/mL), respectively. pUCB group received umbilical cord blood potentiated with rhEPO and rehabilitation

Supporting Information Figure 2. Changes in hemoglobin (A) and erythropoietin (B) levels during the study period of six months According to the protocol, hemoglobin level was monitored at 2 weeks, 3 weeks and 4 weeks after recombinant human erythropoietin (rhEPO) administration, and rhEPO was discontinued, when hemoglobin level reached 15g/dL. Bars represent SE. pUCB group received umbilical cord blood potentiated with recombinant human erythropoietin and rehabilitation; EPO group received recombinant human erythropoietin and rehabilitation; Control group received rehabilitation only.

Supporting Information Figure 3. Baseline differences of glucose metabolism activity with 18F-FDG PET in three groups from paired comparisons (p-value < 0.05) Red colored areas indicate higher glucose metabolism in the later group, and blue colored areas represent lower glucose metabolism in the former group. pUCB group received umbilical cord blood potentiated with recombinant human erythropoietin and rehabilitation; EPO group received recombinant human erythropoietin and rehabilitation; Control group received rehabilitation only.

Supporting Information Figure 4. Comparing changes in fractional anisotropy (FA) from bilateral posterior lower pons, representing spinothalamic tract, during the period from baseline to 6 months post-treatment between three groups; pUCB group (n = 30), EPO group (n = 31), and Control group (n = 29) Bars represent 95% CI. pUCB group received umbilical cord blood potentiated with recombinant human erythropoietin and rehabilitation; EPO group received recombinant human erythropoietin and rehabilitation; Control group received rehabilitation only. p-values are reported for difference between two groups in FA changes, based on post-hoc analysis after ANOVA test.
