# Supplementary material for: Umbilical Cord Blood Therapy Potentiated with Erythropoietin for Children with Cerebral Palsy: A Double-blind, Randomized, Placebo-Controlled Trial
Source: Stem Cells. 2012 Dec 24;31(3):581–91. doi: 10.1002/stem.1304 (PMC3744768; doi:10.1002/stem.1304)
Supplement: Supplementary file 1 [file stem0031-0581-SD1.pdf]

Supporting Information Table 1. Comparison of the duration of the previous rehabilitation, the rehabilitation frequency post-discharge until the study completion, and the residential distribution between three groups

	Group (*n* = 96)		
	**pUCB(*n* =31)**	**EPO(*n* =33)**	**Control(*n* =32)**
**Duration of previous rehabilitation, mean, months***	30.5	36.8	33.1
**The frequency of rehabilitation therapy (per week) post-discharge until completion of study†**			
Physical therapy, mean	5.2	5.6	5.8
Occupational therapy, mean	5.2	5.6	5.6
**The distribution of residence‡, No.**			
Metropolitan cities			
Seoul	2	4	8
Busan	6	3	6
Daegu	0	2	1
Daejeon	1	0	1
Ulsan	2	2	0
Incheon	0	2	3
Province			
Gwangwon-do	3	2	0
Gyeonggi-do	6	12	4
Gyeongsangbuk-do	2	1	1
Gyeongsangnam-do	2	0	4
Chungcheongbuk-do	2	2	0
Chungcheongnam-do	2	2	4
Jeju-do	3	1	0

*† Values are mean and ‡ Values are the numbers of participants.
* The average number of rehabilitation therapy means how many therapies one participant received after discharge until the study completion on weekly basis regarding physical and occupational therapy.
† Duration of previous rehabilitation refers to how long the participants had received rehabilitation treatment before the recruitment.
‡ Residential distribution suggests that participants came from various regions in Korea, and actually the list covers all areas except one metropolitan city and two provinces in Korea.
pUCB group (*n* = 31) received umbilical cord blood potentiated with recombinant human erythropoietin, and rehabilitation; EPO group (*n* = 33) received rhEPO and rehabilitation; Control group (*n* = 32) received rehabilitation only.
None differed significantly between three groups (*p*-value>0.05 for all comparisons)
