# Supplementary material for: Umbilical Cord Blood Therapy Potentiated with Erythropoietin for Children with Cerebral Palsy: A Double-blind, Randomized, Placebo-Controlled Trial
Source: Stem Cells. 2012 Dec 24;31(3):581–91. doi: 10.1002/stem.1304 (PMC3744768; doi:10.1002/stem.1304)
Supplement: Supplementary file 2 [file stem0031-0581-SD2.pdf]

Supporting Information Table 2. Composition of unrelated allogeneic UCB units for pUCB group

Patient	Number of TNC* ($\times 10^7$) per body weight (kg)	Viability (%)	CD34 (+) cells (%)†	Infusion volume‡ (mL)	Number of HLA mismatch§
A1	5.67	95	0.09	17	1¶
A2	5.60	98	0.12	22	1
A3	8.03	90	0.33	20	1
A4	10.53	95	0.22	20	1
A5	5.87	95	0.19	25	1
A6	14.44	86	0.09	15	1
A7	6.17	85	0.29	21	1
A8	6.90	87	0.11	14	1
A9	4.74	90	0.33	20	1
A10	8.38	91	0.29	20	1
A11	16.11	92	0.40	18	1
B1	10.01	98	0.17	20	2**
B2	6.29	94	0.22	25	2
B3	15.29	94	0.21	27	2
B4	8.99	83	0.15	20	2
B5	9.22	95	0.19	25	2
B6	6.69	93	0.37	18	2
B7	5.68	90	0.36	15	2
B8	8.72	90	0.05	17	2
B9	5.95	85	0.13	18	2
B10	17.18	90	0.13	22	2
B11	15.83	93	0.07	17	2
B12	6.12	94	0.75	13	2
B13	5.75	93	0.16	14	2
B14	4.93	86	0.16	15	2
B15	10.42	89	0.15	13	2
B16	5.74	91	0.20	15	2
B17	8.15	92	0.15	20	2
B18	4.34	93	0.25	20	2
B19	4.24	94	0.13	18	2
B20	6.16	93	0.09	11	2

* TNC= total nucleated cells.† CD34 (+) cells (%) was calculated from dividing CD34 (+) cells by TNC in each unit.
‡ Infusion volume of UCB was measured as final volume for the injection, which contained in a syringe after washing.
§ UCB unit matched for four of six HLA types A, B, and DRB1 antigens at least with high resolution.
1¶ and 2** represent one (n = 11) and two (n = 20) mismatched antigens from six HLA antigens.
pUCB group (n = 31) received umbilical cord blood potentiated with recombinant human erythropoietin and rehabilitation.
