# Supplementary material for: Umbilical Cord Blood Therapy Potentiated with Erythropoietin for Children with Cerebral Palsy: A Double-blind, Randomized, Placebo-Controlled Trial
Source: Stem Cells. 2012 Dec 24;31(3):581–91. doi: 10.1002/stem.1304 (PMC3744768; doi:10.1002/stem.1304)
Supplement: Supplementary file 3 [file stem0031-0581-SD3.pdf]

Supporting Information Table 3. Adverse events according to both patient numbers and events within study period of six months

	Group (n = 105)						
	pUCB (n = 35)		EPO (n = 36)		Control (n = 34)		p-value‡
	Patient*	Event†	Patient*	Event†	Patient*	Event†	
**Serious adverse events¶**							
Pneumonia	1	1	2	3	1	1	1.000
Seizure	0	0	1	1	0	0	1.000
Influenza	1	1	0	0	1	1	0.545
Death	1	1	0	0	0	0	0.657
Urinary tract infection	0	0	0	0	1	1	0.324
**Other adverse events**							
Upper respiratory tract infection	18	20	19	23	21	24	0.666
Fever	12	12	4	4	8	8	0.067
Dyspepsia	5	5	2	2	2	2	0.459
Loose stool, diarrhea	6	6	2	2	2	2	0.246
Pneumonia	6	6	0	0	0	0	0.002
Nausea, vomiting	6	6	5	5	2	2	0.398
Anorexia	5	5	2	2	1	1	0.215
Bronchitis	4	4	4	4	3	3	1.000
Constipation	5	5	4	4	5	5	0.878
Irritability	4	4	0	0	0	0	0.021
Febrile convulsion	2	3	1	1	0	0	0.654
Herpangina	0	0	2	2	1	1	0.654
Urticaria	2	2	1	1	4	4	0.254
Hirsuitism	2	2	0	0	0	0	0.212
Seizure	1	1	3	6	3	7	0.625
Alopecia	1	1	0	0	0	0	0.657
Otitis media acute	1	1	1	1	0	0	1.000
Anemia	1	1	0	0	0	0	0.657
Colitis	0	0	1	1	2	2	0.317
Dermatitis	0	0	2	2	2	2	0.465
Insomnia	0	0	1	1	1	1	0.769
Conjunctival injection	0	0	1	1	1	2	0.769
Apnea	3‖	3‖	1	1	1	1	0.527

* Patient denotes the number of patients with adverse events, and †event denotes number of all recorded adverse events, including recurrent adverse events in a same patient. ‡ p-values are reported for differences in number of patients with adverse events between three groups based on Fisher's exact analysis. ¶ Serious adverse events are defined as any events resulted in death, life-threatening situation, hospitalization or prolongation of hospital stay, or otherwise serious consequence by the judgment of the principal investigator. ‖ Apnea in pUCB group means the temporary decline of oxygen saturation at the end of intravenous UCB infusion, and all cases recovered quickly with oxygen supply.
The source of terminology was Medical Dictionary for Regulatory Activities (MedDRA) 14.1.
pUCB group received umbilical cord blood potentiated with recombinant human erythropoietin and rehabilitation; EPO group received recombinant human erythropoietin and rehabilitation; Control group received rehabilitation only.
