# Supplementary material for: Umbilical Cord Blood Therapy Potentiated with Erythropoietin for Children with Cerebral Palsy: A Double-blind, Randomized, Placebo-Controlled Trial
Source: Stem Cells. 2012 Dec 24;31(3):581–91. doi: 10.1002/stem.1304 (PMC3744768; doi:10.1002/stem.1304)
Supplement: Supplementary file 4 [file stem0031-0581-SD4.pdf]

Supporting Information Table 4. Surveyed results of the delayed adverse events post one year from the last participant recruitment

	Group (*n* = 105)		
	**pUCB (*n* = 35)**	**EPO (*n* = 36)**	**Control (*n* = 34)**
**Serious adverse events**[¶]			
Pneumonia	0	0	0
Seizure	0	0	0
Influenza	0	0	0
Death	0	0	0
**Other adverse events**			
Upper respiratory tract infection	0	0	0
Fever	0	0	0
Dyspepsia	0	0	0
Loose stool, diarrhea	1[†]	0	0
Pneumonia	0	0	0
Nausea, vomiting	0	0	0
Anorexia	0	0	0
Bronchitis	0	0	0
Constipation	0	0	0
Irritability	0	0	0
Febrile convulsion	0	0	0
Herpangina	0	0	0
Urticaria	0	0	0
Hirsuitism	1[‡]	0	0
Seizure	0	0	0
Alopecia	0	0	0
Otitis media acute	0	0	0
Anemia	0	0	0
Colitis	0	0	0
Dermatitis	0	0	0
Insomnia	0	0	0
Conjunctival injection	0	0	0

Values are number of patients.

* Serious adverse events are defined as any event, resulted in death, life-threatening situation, hospitalization or prolongation of hospital stay, or otherwise serious consequence by the judgment of the principal investigator.

† One patient was reported to have occasional loose stool, accompanied by antibiotics or diary goods without other enteric symptom.

‡ One patient was reported to have persistent minimal to mild hirsuitism around front hairline, and upper and lower extremities, which lasted about 18 months.

The source of terminology was Medical Dictionary for Regulatory Activities (MedDRA) 14.1.

pUCB group received umbilical cord blood potentiated with recombinant human erythropoietin and rehabilitation; EPO group received recombinant human erythropoietin and rehabilitation; Control group received rehabilitation only.
