# Supplementary material for: Umbilical Cord Blood Therapy Potentiated with Erythropoietin for Children with Cerebral Palsy: A Double-blind, Randomized, Placebo-Controlled Trial
Source: Stem Cells. 2012 Dec 24;31(3):581–91. doi: 10.1002/stem.1304 (PMC3744768; doi:10.1002/stem.1304)
Supplement: Supplementary file 5 [file stem0031-0581-SD5.pdf]

Supporting Information Table 5. Comparison of fractional anisotropy from posterior limbs of internal capsule and spinothalamic tract located in posterior lower pons, and their changes during the period between baseline and 6 months post-treatment

	Point of time for assessment	Groups (n = 90)			*p*-value*	*p*-value†
		pUCB (n = 30)	EPO (n = 31)	Control (n = 29)		
**Posterior limbs of internal capsule**						
Anterior portion, right	Baseline	0.62 (0.02)	0.62 (0.02)	0.58 (0.03)	0.254	
	6-month	0.64 (0.01)	0.64 (0.01)	0.59 (0.03)		
	Difference‡	0.03 (0.01)	0.01 (0.01)	0.01 (0.01)		0.701
	p-value§	0.042§	0.140	0.329		
Posterior Portion, right	Baseline	0.61 (0.02)	0.63 (0.02)	0.60 (0.04)	0.653	
	6-month	0.65 (0.02)	0.66 (0.02)	0.62 (0.03)		
	Difference‡	0.04 (0.01)	0.02 (0.01)	0.02 (0.02)		0.553
	p-value§	0.006§	0.113	0.370		
Anterior Portion, left	Baseline	0.59 (0.02)	0.61 (0.02)	0.56 (0.03)	0.267	
	6-month	0.63 (0.02)	0.63 (0.02)	0.59 (0.03)		
	Difference‡	0.03 (0.01)	0.02 (0.01)	0.04 (0.02)		0.706
	p-value§	0.022§	0.123	0.085		
Posterior Portion, left	Baseline	0.60 (0.02)	0.60 (0.02)	0.57 (0.03)	0.512	
	6-month	0.65 (0.02)	0.64 (0.02)	0.62 (0.03)		
	Difference‡	0.05 (0.02)	0.04 (0.02)	0.05 (0.02)		0.883
	p-value§	0.005§	0.015§	0.036§		
**Posterior lower pons area for spinothalamic tract**						
Right side	Baseline	0.61 (0.01)	0.61 (0.01)	0.59 (0.02)	0.466	
	6-month	0.64 (0.01)	0.61 (0.01)	0.60 (0.02)		
	Difference‡	0.03 (0.01)	0.0 (0.01)	0.01 (0.01)		0.015†
	p-value§	<0.001§	0.820	0.271		
Left side	Baseline	0.62 (0.01)	0.61 (0.01)	0.59 (0.01)	0.223	
	6-month	0.64 (0.01)	0.62 (0.01)	0.59 (0.02)		
	Difference‡	0.02 (0.01)	0.01 (0.01)	0.01 (0.01)		0.404
	p-value§	0.008§	0.287	0.523		

Values are mean (SE).

* *p*-values are reported for difference of fractional anisotropy (FA) between three groups at baseline, based on ANOVA test.

† *p*-values are reported by inter-group analyses for differences of FA change during the period between baseline and 6-month post-treatment, based on ANOVA test.

pUCB group received umbilical cord blood potentiated with recombinant human erythropoietin and rehabilitation; EPO group received recombinant human erythropoietin and rehabilitation; Control group received rehabilitation only.

§ *p*-values are reported by intra-group analyses for FA changes from baseline to 6-month post-treatment, based on paired t-test. Significant *p*-values (<0.05) are marked with the same symbol.

‡ Difference represents mean value of subtracting FA assessed at baseline, from FA at 6 month post-treatment in each group.
