# Supplementary material for: Umbilical Cord Blood Therapy Potentiated with Erythropoietin for Children with Cerebral Palsy: A Double-blind, Randomized, Placebo-Controlled Trial
Source: Stem Cells. 2012 Dec 24;31(3):581–91. doi: 10.1002/stem.1304 (PMC3744768; doi:10.1002/stem.1304)
Supplement: Supplementary file 6 [file stem0031-0581-SD6.pdf]

Supporting Information Table 6. Changes in the functional assessment values during intervals between assessment times in each group

		Assessment time	Assessment times for comparison	pUCB (n = 31)		EPO (n = 33)		Control (n = 32)	
				Values	*p*-value*	Values	*p*-value*	Values	*p*-value*
**GMPM**		Baseline		34.5 (2.7)		38.2 (2.8)		35.5 (2.9)	
		1 month	0–1 month	41.5 (3.0)	<0.001	42.7 (2.6)	<0.001	41.6 (3.1)	<0.001
		3 months	1–3 month	46.0 (3.0)	<0.001	45.7 (2.7)	<0.001	43.6 (3.1)	0.001
		6 months	3–6 month	49.0 (3.1)	<0.001	47.4 (2.7)	0.001	45.1 (3.2)	0.001
**BSID-II Mental scale raw score**		Baseline		94.1 (9.1)		105.8 (8.9)		86.6 (9.0)	
		1 month	0–1 month	102.4 (9.1)	<0.001	109.3 (8.9)	<0.001	89.9 (9.0)	<0.001
		3 months	1–3 month	106.1 (8.9)	<0.001	113.3 (8.9)	<0.001	92.4 (9.1)	0.001
		6 months	3–6 month	111.7 (9.0)	<0.001	117.4 (8.8)	<0.001	96.5 (9.2)	<0.001
**BSID-II Motor scale raw score**		Baseline		45.2 (4.7)		53.3 (5.0)		45.5 (4.6)	
		1 month	0–1 month	50.3 (4.1)	0.002	58.4 (5.2)	0.022	48.2 (4.7)	<0.001
		3 months	1–3 month	54.8 (4.3)	<0.001	60.0 (5.1)	0.002	49.7 (4.7)	0.001
		6 months	3–6 month	56.9 (4.5)	0.002	60.9 (5.1)	0.035	50.7 (4.8)	0.010
**GMFM**		Baseline		36.0 (4.7)		42.5 (4.9)		36.8 (4.5)	
		1 month	0–1 month	39.7 (4.9)	<0.001	46.8 (5.1)	<0.001	41.5 (4.7)	<0.001
		3 months	1–3 month	42.6 (4.8)	0.001	49.4 (5.1)	<0.001	43.3 (4.7)	<0.001
		6 months	3–6 month	45.1 (4.9)	<0.001	51.6 (5.1)	0.002	44.6 (4.8)	0.001
**PEDI Functional skills scale**	Self care	Baseline		39.1 (2.5)		38.2 (2.7)		37.4 (2.2)	
		1 month	0–1 month	42.2 (2.2)	<0.001	40.4 (2.9)	<0.001	40.0 (2.2)	<0.001
		3 months	1–3 month	44.7 (2.4)	<0.001	43.5 (2.9)	<0.001	41.7 (2.3)	<0.001
		6 months	3–6 month	46.5 (2.5)	<0.001	45.0 (2.8)	<0.001	42.8 (2.3)	0.001
	Mobility	Baseline		24.8 (3.7)		29.2 (4.3)		24.9 (3.7)	
		1 month	0–1 month	28.1 (3.9)	0.001	32.4 (4.3)	<0.001	27.9 (3.8)	<0.001
		3 months	1–3 month	31.7 (4.3)	0.002	34.6 (4.3)	<0.001	29.3 (3.9)	
		6 months	3–6 month	34.6 (4.3)	<0.001	36.6 (4.4)	0.002	31.4 (3.9)	<0.001
	Social function	Baseline		40.0 (3.5)		40.1 (3.3)		34.8 (3.5)	
		1 month	0–1 month	42.8 (3.6)	<0.001	42.5 (3.3)	<0.001	37.5 (3.6)	0.011
		3 months	1–3 month	46.4 (3.9)	0.001	45.8 (3.3)	<0.001	39.6 (3.5)	0.004
		6 months	3–6 month	49.1 (3.6)	<0.001	47.8 (3.4)	0.001	42.3 (3.9)	0.006
**PEDI Caregiver assistance**	Self care	Baseline		18.0 (4.1)		17.9 (3.7)		15.3 (3.2)	
		1 month	0–1 month	20.1 (4.0)	0.097	21.1 (3.8)	0.030	19.0 (3.7)	0.015
		3 months	1–3 month	27.0 (4.3)	<0.001	24.2 (3.9)		21.5 (3.7)	0.023
		6 months	3–6 month	29.4 (4.4)	0.017	26.0 (4.1)		23.1 (3.9)	0.002
	Mobility	Baseline		14.6 (4.1)		19.7 (4.8)		13.5 (4.0)	
		1 month	0–1 month	19.6 (4.5)	0.002	22.6 (4.9)	0.008	15.6 (4.2)	
		3 months	1–3 month	22.5 (5.1)		26.0 (5.3)	0.006	19.3 (4.5)	0.028
		6 months	3–6 month	24.9 (5.5)	0.018	29.2 (5.4)	0.025	24.0 (4.8)	0.007
	Social function	Baseline		23.0 (5.2)		24.7 (3.9)		18.2 (4.4)	
		1 month	0–1 month	28.5 (5.6)	<0.001	29.6 (4.5)	<0.001	21.1 (4.8)	0.015
		3 months	1–3 month	34.9 (6.0)	0.001	34.2 (4.8)	<0.001	24.2 (5.0)	0.023
		6 months	3–6 month	38.4 (6.2)	0.006	36.2 (4.6)		27.6 (5.3)	
**WeeFIM**	Total score	Baseline		34.1 (3.8)		36.6 (3.7)		31.4 (2.9)	
		1 month	0–1 month	35.6 (4.1)	0.019	37.5 (3.8)	<0.001	33.2 (3.1)	0.008
		3 months	1–3 month	38.7 (4.7)	0.002	38.5 (4.1)		36.5 (3.7)	0.007
		6 months	3–6 month	41.1 (4.8)	0.006	39.6 (4.1)	0.009	37.3 (3.8)	0.041
	Self care	Baseline		9.3 (1.0)		9.6 (0.9)		8.4 (0.7)	
		1 month	0–1 month	9.7 (1.1)		9.7 (0.9)		9.3 (0.8)	0.038
		3 months	1–3 month	10.4 (1.2)	0.010	10.2 (1.0)	0.021	9.8 (0.9)	0.045
		6 months	3–6 month	11.0 (1.3)	0.048	10.5 (1.0)	0.012	9.9 (0.9)	
	Sphincter control	Baseline		4.7 (0.8)		4.6 (0.7)		4.2 (0.6)	
		1 month	0–1 month	4.8 (0.8)		4.7 (0.7)		4.2 (0.6)	
		3 months	1–3 month	5.1 (0.9)		4.8 (0.7)		4.8 (0.8)	
		6 months	3–6 month	5.3 (0.9)		5.1 (0.7)		4.9 (0.8)	
	Mobility	Baseline		5.4 (0.9)		6.2 (1.0)		5.1 (0.7)	
		1 month	0–1 month	5.6 (0.9)		6.3 (1.0)		5.3 (0.7)	
		3 months	1–3 month	6.3 (1.0)	0.008	6.5 (1.0)		6.5 (0.9)	
		6 months	3–6 month	6.6 (1.0)	0.090	6.5 (1.0)		6.9 (0.9)	
	Locomotion	Baseline		4.1 (0.5)		4.7 (0.5)		3.8 (0.4)	
		1 month	0–1 month	4.3 (0.5)	0.057	5.0 (0.6)	0.030	4.1 (0.4)	0.017
		3 months	1–3 month	4.7 (0.6)	0.050	5.2 (0.6)		4.5 (0.5)	
		6 months	3–6 month	5.1 (0.6)	0.032	5.2 (0.6)		4.8 (0.6)	
	Communicatio n	Baseline		4.6 (0.6)		5.3 (0.7)		4.3 (0.6)	
		1 month	0–1 month	5.0 (0.6)	0.032	5.5 (0.7)		4.6 (0.6)	0.027
		3 months	1–3 month	5.4 (0.7)	0.017	5.6 (0.7)		4.8 (0.7)	
		6 months	3–6 month	5.9 (0.8)	0.020	5.8 (0.7)	0.030	4.8 (0.6)	
	Social cognition	Baseline		5.9 (0.7)		6.4 (0.7)		5.6 (0.6)	
		1 month	0–1 month	6.1 (0.8)		6.5 (0.7)	0.057	5.8 (0.7)	
		3 months	1–3 month	6.8 (0.9)	0.022	6.4 (0.7)		6.1 (0.8)	0.039
		6 months	3–6 month	7.2 (0.9)	0.024	6.5 (0.7)		6.0 (0.7)	
**Summation of MMT**		Baseline		100.7 (5.5)		104.8 (4.4)		100.3 (4.6)	
		1 month	0–1 month	105.0 (4.9)	0.008	106.8 (4.5)	0.173	101.8 (4.6)	0.019
		3 months	1–3 month	107.8 (4.4)	0.016	107.7 (4.5)	0.005	103.6 (4.6)	0.010
		6 months	3–6 month	109.9 (4.6)	0.033	109.3 (4.5)	0.130	104.3 (4.6)	0.012

Values are mean (SE). There was no significant difference in all baseline values between three groups. *p-values are reported only for significant difference of outcome scores during each interval of assessments, such as baseline to 1-month (0-1 month), 1-month to 3-month (1-3 month), and 3-month to 6-month (3-6 month) post-treatment within group, based on paired t-test
