# Supplementary material for: Umbilical Cord Blood Therapy Potentiated with Erythropoietin for Children with Cerebral Palsy: A Double-blind, Randomized, Placebo-Controlled Trial
Source: Stem Cells. 2012 Dec 24;31(3):581–91. doi: 10.1002/stem.1304 (PMC3744768; doi:10.1002/stem.1304)
Supplement: Supplementary file 7 [file stem0031-0581-SD7.pdf]

Supporting Information Table 7. Comparison of differences in outcome between three groups, respectively in the younger than 36 month-old and in the 36 month-old or older

	Interval between assessments	Younger than 36 months old group (*n* = 45)				36 months old or older group (*n* = 51)			
		pUCB (*n*=16)	EPO (*n*=13)	Control (*n*=16)	*p*-value*†‡	pUCB (*n*=15)	EPO (*n*=20)	Control (*n*=16)	*p*-value†
**GMPM**	0–1month	5.3(1.2)	6.5(1.1)	5.9(1.6)		8.8(2.3)	3.2(0.7)	6.2(1.3)	
	0–3month	10.0(1.3)	9.6(1.4)	8.3(1.7)		13.0(2.9)	6.1(0.7)	7.8(1.7)	
	0–6month	14.0(1.9)	12.0(1.2)	9.5(1.7)		15.1(3.1)	7.4(0.9)	9.7(1.8)	
	1–3month	4.8 (0.8)	3.1(0.8)	2.4(0.8)		4.2(1.2)	2.8(0.7)	1.7(0.9)	
	1–6month	8.8(1.2)	5.5(1.2)	3.6(1.0)	0.010†	6.2(1.3)	4.2(0.9)	3.5(1.1)	
	3–6month	4.0(0.8)	2.4(1.1)	1.2(0.7)		2.1(0.7)	1.3(0.4)	1.8(0.4)	
**BSID-II Mental scale raw score**	0–1month	9.4(2.0)	3.8(0.9)	2.9(0.7)	0.013†	7.0(1.7)	3.2(0.6)	3.8(0.8)	
	0–3month	12.7(2.2)	9.3(1.3)	6.1(1.2)	0.046†	11.3(1.9)	6.2(1.1)	5.5(1.2)	0.031†
	0–6month	18.8(2.8)	14.8(2.2)	10.1(2.5)		16.4(2.3)	9.4(1.4)	9.6(2.0)	
	1–3month	3.3(0.9)	5.5(1.1)	3.3(0.9)		4.3(1.4)	3.0(1.0)	1.8(1.0)	
	1–6month	9.4(1.6)	11.0(2.2)	7.3(2.1)		9.4(2.4)	6.2(1.4)	5.9(1.8)	
	3–6month	6.1(1.6)	5.5(1.3)	4.0(1.5)		5.1(1.8)	3.2(0.9)	4.1(1.5)	
**BSID-II Motor scale raw score**	0–1month	3.3(0.8)	3.2(1.0)	2.1(0.8)		6.9(2.9)	3.1(0.8)	3.3(0.9)	
	0–3month	8.3(2.0)	5.8(1.0)	3.0(0.9)	0.028†	10.9(3.2)	4.1(1.0)	5.5(1.2)	
	0–6month	11.0(2.5)	6.4(1.0)	4.1(1.3)	0.039†	12.5(3.2)	5.2(1.2)	6.3(1.2)	
	1–3month	5.0(1.6)	2.5(0.6)	0.9(0.5)		4.0(1.4)	1.0(0.7)	2.2(0.6)	
	1–6month	7.7(2.1)	3.2(0.7)	2.0(0.8)	0.028†	5.6(1.5)	2.1(0.9)	3.0(0.8)	
	3–6month	2.7(1.0)	0.6(0.4)	1.1(0.6)		1.6(0.7)	1.1(0.6)	0.8(0.4)	
**GMFM**	0–1month	2.8(0.4)	4.4(0.9)	3.7(0.7)		4.5(0.7)	4.2(0.7)	5.6(0.9)	
	0–3month	6.7(1.7)	8.4(1.5)	5.9(0.9)		6.4(0.8)	5.8(0.7)	6.9(1.1)	
	0–6month	10.6(2.2)	12.7(2.1)	7.1(1.2)		7.5(0.9)	6.6(0.8)	8.4(1.4)	
	1–3month	3.9(1.5)	4.0(1.2)	2.3(0.6)		1.8(0.3)	1.6(0.3)	1.3(0.4)	
	1–6month	7.8(2.1)	8.3(1.9)	3.5(0.9)		3.0(0.5)	2.4(0.4)	2.8(0.9)	
	3–6month	3.9(0.7)	4.3(1.4)	1.2(0.4)	0.005†‡	1.1(0.3)	0.8(0.2)	1.5(0.6)	

Values are mean (SE).

GMPM denotes Gross Motor Performance Measure; BSID-II, Bayley Scales of Infant Development, 2nd edition; GMFM, Gross Motor Function Measure.

pUCB group received umbilical cord blood potentiated with recombinant human erythropoietin and rehabilitation; EPO group received recombinant human erythropoietin and rehabilitation; Control group received rehabilitation only.

p-values are reported for difference of outcome changes between three groups during each interval, based on the Kruskal-Wallis test.

*, † or ‡ were marked if *p*-values are significant (<0.05), and * means pUCB group > EPO group while † means pUCB group > Control group and ‡ refers to EPO group > Control group after post-hoc analysis.
