# Supplementary material for: Umbilical Cord Blood Therapy Potentiated with Erythropoietin for Children with Cerebral Palsy: A Double-blind, Randomized, Placebo-Controlled Trial
Source: Stem Cells. 2012 Dec 24;31(3):581–91. doi: 10.1002/stem.1304 (PMC3744768; doi:10.1002/stem.1304)
Supplement: Supplementary file 9 [file stem0031-0581-SD9.pdf]

Supporting Information Table 9. Comparison of differences in outcome between three groups, respectively in the less impaired as GMFCS levels I–III groups and in the more impaired as GMFCS IV and V groups

	Interval between assessments	Less impaired as GMFCS I–III (*n* = 47)				More impaired as GMFCS IV, V (*n* = 49)			
		pUCB (*n*=12)	EPO (*n*=20)	Control (*n*=15)	*p*-value*†	pUCB (*n*=19)	EPO (*n*=13)	Control (*n*=17)	*p*-value*†
**GMPM**	0–1month	6.6(2.2)	5.1(0.9)	6.4(1.3)		7.2(1.7)	3.6(1.0)	5.7(1.6)	
	0–3month	10.8(2.3)	8.6(1.0)	8.5(1.7)		11.9(2.1)	5.7(0.9)	7.7(1.7)	
	0–6month	15.5(3.0)	10.5(1.0)	10.3(1.7)		13.9(2.2)	7.1(1.2)	8.9(1.8)	
	1–3month	4.1(0.9)	3.5(0.7)	2.1(1.1)		4.7(1.0)	2.1(0.8)	2.0(0.6)	0.045
	1–6month	8.9(1.5)	5.4(0.9)	4.0(1.2)	0.021†	6.7(1.2)	3.5(1.2)	3.2(0.9)	
	3–6month	4.7(1.0)	1.9(0.7)	1.9(0.4)	0.035*	2.0(0.6)	1.4(0.7)	1.2(0.7)	
**BSID-II Mental scale raw score**	0–1month	8.3(1.2)	4.1(0.7)	3.9(0.7)	0.010*†	8.2(2.1)	2.5(0.6)	2.8(0.8)	
	0–3month	11.1(1.2)	9.3(1.0)	6.9(1.1)		12.6(2.3)	4.6(1.3)	4.9(1.2)	0.010*†
	0–6month	17.3(1.6)	14.3(1.6)	10.9(2.0)		17.8(2.8)	7.3(1.5)	8.9(2.4)	0.017†
	1–3month	2.8(0.9)	5.2(0.9)	2.9(1.0)		4.4(1.2)	2.2(1.1)	2.1(1.0)	
	1–6month	9.0(1.8)	10.2(1.7)	7.0(1.8)		9.6(2.0)	4.8(1.4)	6.2(2.1)	
	3–6month	6.2(1.6)	5.0(1.1)	4.1(1.2)		5.3(1.7)	2.7(0.8)	4.1(1.7)	
**BSID-II Motor scale raw score**	0–1month	3.0(0.9)	4.1(0.8)	3.2(1.0)		6.3(2.3)	1.8(0.8)	2.3(0.7)	
	0–3month	7.6(2.4)	5.8(1.0)	4.1(1.1)		10.8(2.7)	3.2(1.1)	4.4(1.1)	0.041*
	0–6month	11.3(2.5)	7.1(1.0)	5.3(1.2)		11.9(2.8)	3.4(1.0)	5.1(1.4)	0.030*
	1–3month	4.6(1.8)	1.8(0.7)	0.9(0.2)		4.5(1.3)	1.4(0.7)	2.1(0.7)	
	1–6month	8.3(2.0)	3.1(0.8)	2.1(0.6)	0.016†	5.6(1.7)	1.6(0.7)	2.8(0.9)	
	3–6month	3.8(1.0)	1.3(0.6)	1.2(0.5)	0.033*	1.2(0.8)	0.2(0.2)	0.8(0.5)	
**GMFM**	0–1month	3.7(0.5)	5.7(0.6)	5.9(0.9)		3.6(0.6)	2.1(0.4)	3.6(0.6)	
	0–3month	8.1(2.1)	8.8(1.0)	8.3(1.1)		5.5(0.7)	3.8(0.5)	4.8(0.8)	
	0–6month	12.0(2.8)	11.7(1.4)	9.9(1.4)		7.2(0.8)	4.9(0.6)	5.9(1.0)	
	1–3month	4.4(2.0)	3.1(0.9)	2.4(0.7)		1.9(0.3)	1.7(0.4)	1.3(0.4)	
	1–6month	8.3(2.8)	6.0(1.4)	4.0(1.0)		3.6(0.5)	2.8(0.5)	2.4(0.7)	
	3–6month	3.9(0.9)	2.9(1.0)	1.6(0.6)		1.7(0.4)	1.2(0.4)	1.1(0.5)	

Values are mean (SE).

GMPM denotes Gross Motor Performance Measure; BSID-II, Bayley Scales of Infant Development, 2nd edition; GMFM, Gross Motor Function Measure.

pUCB group received umbilical cord blood potentiated with recombinant human erythropoietin and rehabilitation; EPO group received recombinant human erythropoietin and rehabilitation; Control group received rehabilitation only.

p-values are reported for difference of outcome changes between three groups during each interval, based on the Kruskal-Wallis test.

*, † or ‡ were marked if *p*-values are significant (<0.05), and * means pUCB group > EPO group while † means pUCB group > Control group and ‡ refers to EPO group > Control group after post-hoc analysis. *p*-values without symbols indicate that there was no significant difference in post-hoc analysis.
