# Supplementary material for: Umbilical Cord Blood Therapy Potentiated with Erythropoietin for Children with Cerebral Palsy: A Double-blind, Randomized, Placebo-Controlled Trial
Source: Stem Cells. 2012 Dec 24;31(3):581–91. doi: 10.1002/stem.1304 (PMC3744768; doi:10.1002/stem.1304)
Supplement: Supplementary file 12 [file stem0031-0581-SD12.pdf]

Supporting Information Table 12. Clinical outcomes and Total Nucleated Cells (TNC) and CD34+ cells per kg of body weight in pUCB group (*n* = 31)

**TNC number/kg of body weight**		**≥ 6.69 × 10^7 (*n* = 16)**	**< 6.69 × 10^7 (*n* = 15)**	***p*-value***
GMFM	3–6month	3.6 (0.8)	1.5 (0.3)	0.042
	1–6month	7.6 (2.1)	3.1 (0.5)	0.038
GMPM	1–3month	5.2 (0.6)	3.7 (1.3)	0.038
**CD 34+ cell number/kg of body weight**		**≥ 1.46 × 10^5 (*n* = 16)**	**< 1.46 × 10^5 (*n* = 15)**	***p*-value***
BSID-II Mental scale raw score	3–6month	8.3 (1.9)	2.7 (0.9)	0.029
	1–6month	13.2 (2.2)	5.3 (0.9)	0.009

Values are mean (SE).

GMFM denotes Gross Motor Performance Measure; GMPM, Gross Motor Performance Measure; BSID-II, Bayley scales of infant development, 2nd edition

For TNC/kg, 6.69 x 10^7 was median value and for CD34+ cell numbers/kg, 1.46 x 10^5 was median value.

pUCB group received umbilical cord blood potentiated with recombinant human erythropoietin and rehabilitation.

* *p*-values are reported for difference between two groups, based on Mann-Whitney analysis.
