# Supplementary material for: Umbilical Cord Blood Therapy Potentiated with Erythropoietin for Children with Cerebral Palsy: A Double-blind, Randomized, Placebo-Controlled Trial
Source: Stem Cells. 2012 Dec 24;31(3):581–91. doi: 10.1002/stem.1304 (PMC3744768; doi:10.1002/stem.1304)
Supplement: Supplementary file 13 [file stem0031-0581-SD13.pdf]

Supporting Information Table 13-A. Areas of increased glucose metabolism in three groups (*p*-value < 0.05), obtained by SPM analysis of ^{18}F-FDG-PET/CT scan

Group	Coordinate				Functional area	Voxel level	
	x	y	z			Z	$P_{uncorrected}$
pUCB	30	-6	-4	Rt	Lentiform nucleus, gray matter, putamen	2.89	0.002
	-14	12	-18	Lt	Frontal lobe, medial frontal gyrus	2.71	0.003
	44	-12	14	Lt	Sub-lobar, insula	2.51	0.005
	-24	4	2	Lt	Lentiform nucleus, gray matter, putamen	2.8	0.003
	-22	-12	4	Lt	Lentiform nucleus, gray matter, lateral globus pallidus	2.58	0.004
	-10	-28	-8	Lt	Thalamus; Midbrain	2.44	0.007
	12	-40	52	Rt	Parietal lobe, precuneus	2.33	0.010
	-64	-14	-12	Lt	Temporal lobe, middle temporal gyrus	2.32	0.010
	10	-32	64	Rt	Parietal lobe, precentral and postcentral gyrus	1.98	0.024
EPO	10	-24	48	Rt	Frontal lobe, paracentral lobule	3.72	< 0.001
	8	32	14	Rt	Limbic lobe, anterior cingulate	3.51	< 0.001
	-40	-20	36	Lt	Frontal lobe, precentral gyrus	3.01	0.001
	-25	8	8	Lt	Lentiform nucleus, putamen	2.83	0.002
Control	0	-38	-18	Rt	Cerebellum, anterior lobe, culmen	3.54	< 0.001
	-12	-50	-32	Lt	Cerebellum, posterior lobe, cerebellar tonsil	3.32	< 0.001
	12	24	-20	Rt	Frontal lobe, orbital gyrus	2.04	0.021

pUCB group received umbilical cord blood potentiated with recombinant human erythropoietin and rehabilitation; EPO group received recombinant human erythropoietin and rehabilitation; Control group received rehabilitation only.
The data were then normalized to a standard PET template provided by SPM8.
