# Supplementary material for: Umbilical Cord Blood Therapy Potentiated with Erythropoietin for Children with Cerebral Palsy: A Double-blind, Randomized, Placebo-Controlled Trial
Source: Stem Cells. 2012 Dec 24;31(3):581–91. doi: 10.1002/stem.1304 (PMC3744768; doi:10.1002/stem.1304)
Supplement: Supplementary file 14 [file stem0031-0581-SD14.pdf]

Supporting Information Table 13-B. Areas of decreased glucose metabolism in three groups (*p*-value < 0.05), obtained by SPM analysis of ^{18}F-FDG-PET/CT scan

Group	Coordinate				Functional area	Voxel level	
	x	y	z			Z	*P* uncorrected
pUCB	28	-80	6	Rt	Occipital lobe, middle cccipital gyrus	3.66	< 0.001
	-25	-55	0	Lt	Limbic lobe, parahippocampal gyrus	3.09	0.001
	22	-50	-2	Rt	Limbic lobe, parahippocampal gyrus	2.94	0.002
	-28	-12	40	Lt	Frontal lobe, middle frontal gyrus	1.75	0.04
	48	-54	0	Rt	Temporal lobe, inferior temporal gyrus	1.73	0.041
	-40	-30	62	Lt	Parietal lobe, postcentral gyrus	1.73	0.042
	-8	-94	-26	Lt	Posterior lobe, uvula	1.7	0.044
	-30	-8	36	Lt	Frontal lobe, precentral gyrus	1.67	0.048
	46	-50	-4	Rt	Temporal lobe, sub-gyral	1.67	0.048
EPO	40	-66	-20	Rt	Occipital lobe; posterior lobe, declive	3.81	< 0.001
	45	-48	-25	Rt	Occipital lobe; Anterior lobe, culmen	3.52	< 0.001
	-42	-55	-22	Lt	Occipital lobe; Posterior lobe, declive	3.43	< 0.001
Control	-28	42	-4	Lt	Frontal lobe, middle frontal gyrus	3.27	0.001
	-38	8	2	Lt	Sub-lobar, insula	2.22	0.013
	34	38	18	Rt	Frontal lobe, middle frontal gyrus	3.05	0.001
	34	42	-2	Rt	Frontal lobe, sub-gyral	3.03	0.001
	-48	-46	-18	Lt	Temporal lobe, fusiform gyrus	3.00	0.001
	-50	-35	-18	Lt	Temporal lobe, inferior temporal gyrus	2.91	0.002
	-50	-46	0	Lt	Temporal lobe, middle temporal gyrus	2.74	0.003
	55	-2	-25	Rt	Temporal lobe, middle temporal gyrus	2.13	0.022
	10	28	-4	Rt	Limbic lobe, anterior cingulate	2.04	0.021

pUCB group received umbilical cord blood potentiated with recombinant human erythropoietin and rehabilitation; EPO group received recombinant human erythropoietin and rehabilitation; Control group received rehabilitation only.
The data were then normalized to a standard PET template provided by SPM8.
