# Supplementary material for: Umbilical Cord Blood Therapy Potentiated with Erythropoietin for Children with Cerebral Palsy: A Double-blind, Randomized, Placebo-Controlled Trial
Source: Stem Cells. 2012 Dec 24;31(3):581–91. doi: 10.1002/stem.1304 (PMC3744768; doi:10.1002/stem.1304)
Supplement: Supplementary file 15 [file stem0031-0581-SD15.pdf]

Supporting Information

Supporting information to: Umbilical Cord Blood Therapy Potentiated with Erythropoietin for Children with Cerebral Palsy: A Double-blind, Randomized, Placebo-controlled Trial

Contents:

1. Accomplishment of the double-blinded, randomized placebo-controlled trial………..… 2

Study settings

Intervention

The drop-out cases

Completion of study and breaking concealment

2. Efficacy measurement ….……………......………………………………………......……….… 2

Measurement for neurodevelopmental function scores

Measurement of fractional anistropy

Contents 1. Accomplishment of the double-blinded, randomized placebo-controlled trial

Study settings: This study was a single center trial at the Rehabilitation Medicine Center in CHA Bundang Hospital, a University Hospital, located at highly populated south east suburb of Seoul. The institute supplied comprehensive rehabilitation medical approaches for all kinds of disabilities, including brain injures in all ages. Ten pediatric physical therapists and ten pediatric occupational therapists undertook the assessments and treatments to the participants of this trial. All were trained for the assessments before the trial and tested for reliabilities from the main outcomes, and all were specialized for pediatric rehabilitations with experiences of more than 18 months. All participants received an intensive 1-month in-patient rehabilitation program, consisting of two sessions of physical and occupational therapy per day. The rehabilitative therapy sessions were individualized and lasted 30-minutes per session. The purpose of rehabilitation therapy was to facilitate normal movements and reactions by inducing active participations in the therapeutic intervention, providing opportunities to experience various sensory inputs, and inducing cognitive and perceptual stimulations. All parents or care providers of the participants were educated for home exercise programme and therapeutic playing with their children. After discharge each participant continued to receive rehabilitation therapy at least three days per week until the last follow-up assessments at 6 months post-treatment.

Intervention: High-resolution HLA and blood type matches were evaluated before randomization procedure in the study. All cord blood units were obtained from the donors, who provided written informed consent for the donation of their UCB to the cord blood bank and were examined for any infection.

Each UCB unit was washed for infusion by the protocol of Rubinstein followed by a viability assessment and TNC counts to confirm the previous information. For the umbilical cord blood infusion, only the principal investigator conducted the procedure. The 22-gauge needles were used for the infusion, and prepared peripheral veins were used. A single intravenous infusion was administered to each subject, and their vital signs and oxygen levels at saturation were monitored in a room equipped for emergencies. The infusion duration was about 3 minutes, and it took about 30 ~ 40 minutes for each injection including preparation, monitoring oxygen saturation, blood pressure, heart rate, and observation for stable vital status after injection.

For pUCB group, the dosage of cyclosporine was adjusted to maintain the blood level within the appropriate range (approximately 100–200 ng/mL) by monitoring blood samples at 1, 3, and 5 days after the infusion, followed by once per week schedule for 3 weeks. The administration of cyclosporine was repeated as needed.

The drop-out cases: There were nine drop outs: one death; six withdrawals from the follow-up assessment; and two ineligible cases, one due to a surgical procedure for hydrocephalus and the other one due to a history of erythropoietin treatment during infancy. Sorting exact reasons for the six arbitrary withdrawal by the parents was difficult due to limited explanations from their parents; in two cases (one in pUCB, and one in EPO group), parents seemed to fall into despair and lost motivations of participating in the trial to the completion; the parents of two participants in EPO group seemed to be annoyed to undertake the follow-up assessments; and the parents of last two participants (one in pUCB and one in Control group) seemed to have family problems, caused difficulties in the follow-ups. One girl with previously diagnosed

hydrocephalus dropped out because of surgical procedure for hydrocephalus. The decision for the surgical therapy was made with a different opinion from a newly introduced neurosurgeon, whom saw no change in brain MRI finding or clinical aggravation after UCB infusion. One participant was excluded due to ineligibility from the previous rhEPO treatment during infancy, which was revealed by chance after finishing the group inclusion and treatment. And, there was one mortality case. She showed the poorest motor function among the participants and had no head control in addition to the constant presence of obstructive phlegm. Tube feeding was required due to poor oral motor function; however, her parents insisted on oral feeding. She had been medically stable post-treatment with continuous neurological improvement up until the 3-months follow-up evaluation, and her movement was video-recorded. When she visited the pediatric neurology department for the routine follow-up of seizures on the day of her death, she was found to be neurologically stable. The patient did not show any signs of functional decline, seizure aggravation, or any laboratory abnormalities. Her death occurred during sleep without any apparent cause. The death was regarded as caused by obstructive respiratory arrest from saliva or regurgitated food. She had quadriplegic cerebral palsy with spasticity from profound hypoxia with involvement of the central gray matter and brain stem, as shown on MRI. Her brain MRI per se seemed to have high risk of mortality, as shown below.

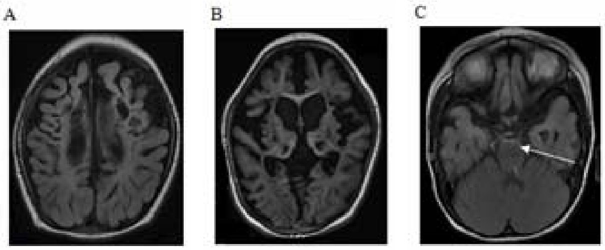

Brain MRI finding of one mortality case, undertaken in September, 2010, at enrollment to this study
A. Multiple encephalomalatic changes were observed in bilateral frontal lobes, including cortical gray matter
B. Diffuse brain atrophy with encephalomalacia was observed in basal ganglia indicated profound hypoxic ischemic encephalopathy in deep nuclear structures
C. Diffuse atrophy of pons (white arrow)

In the mortality case, there was no hypertension during cyclosporine administration. Hypertension is one of symptoms of PRES (Posterior Reversible Encephalopathy Syndrome) with other symtoms such as seizure, headache, mental abnormalities, and abnormalities of visual perception. (*Endo A, Fuchigami T, Hasegawa M, et al. Posterior reversible encephalopathy syndrome in childhood: report of four cases and review of the literature. Pediatr Emerg Care 2012;28:153-157*)

<u>Completion of study and breaking the concealment</u>: This trial was purposed to monitor each participant for six months and it was completed as purposed. No interim analysis was conducted. For the serious adverse events, those were reported to the principal investigator. And the principal investigator reported the cases to Safety Monitoring Board (SMB) within designated days. The trial was designed to be stopped, if continuation might prove harmful to participants, based on the judgment by SMB or the principal investigator. In case of one mortality in the pUCB group, the incidence was categorized, as "unlikely possible" with approval after the examination by SMB, and the trial continued. Total breaking of the concealment was conducted at the completion of the trial, and the principal investigator opened the assignment after receiving the information from the cord blood provider.

Contents 2. Efficacy measurements

<u>Measurement for neurodevelopmental function scores</u>

For the functional measurements, all previously verified Korean version or authorized original version of assessment tools were used. Pre-trial reliability tests were conducted for the main outcomes.

The result of manual muscle strength test was converted into scores (MMT: zero, 0; trace, 1; poor, 2; fair, 3; good, 4; and normal, 5) for flexors, extensors, abductors, and adductors of the bilateral shoulder and hip joints; flexors and extensors of the bilateral elbow, wrist, and knee; and dorsiflexors and plantar flexors of the ankles (full score, 160).

Measurement of fractional anisotrop

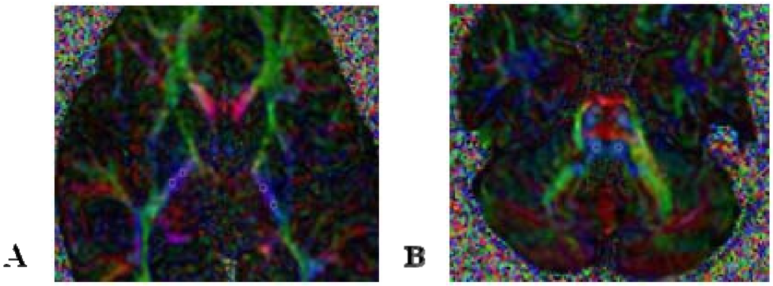

Designated regions of interest are marked with white circles for measuring the fractional anisotropy at (A) four loci in bilateral posterior limbs of internal capsule, as anterior and posterior portions, and (B) two loci in bilateral posterior lower pons.
