# Supplementary figures and images for: Umbilical Cord Blood Therapy Potentiated with Erythropoietin for Children with Cerebral Palsy: A Double-blind, Randomized, Placebo-Controlled Trial
Source: Stem Cells. 2012 Dec 24;31(3):581–91. doi: 10.1002/stem.1304 (PMC3744768; doi:10.1002/stem.1304)

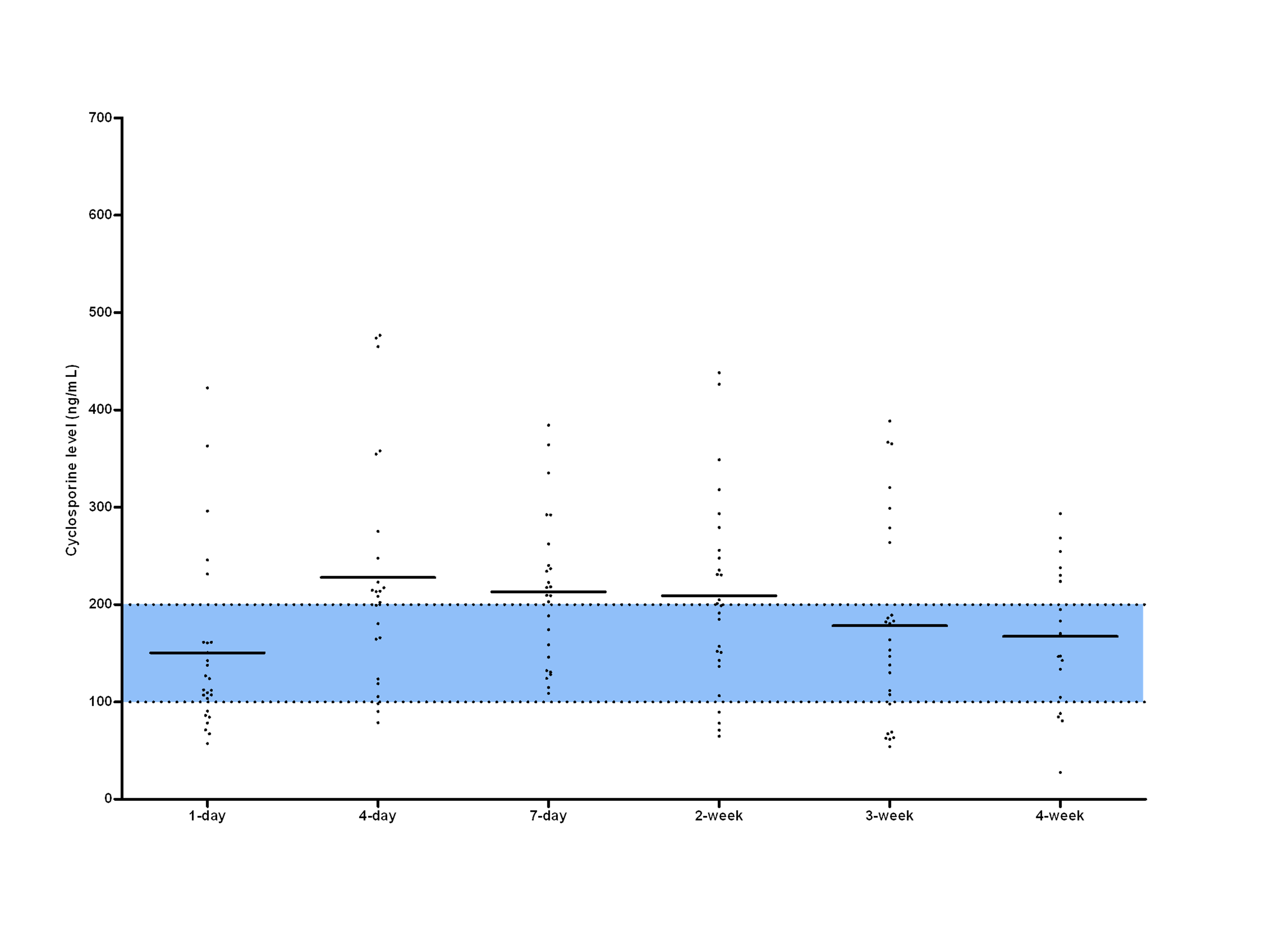

Supplement: Supplementary file 16 [file stem0031-0581-SD16.tif]

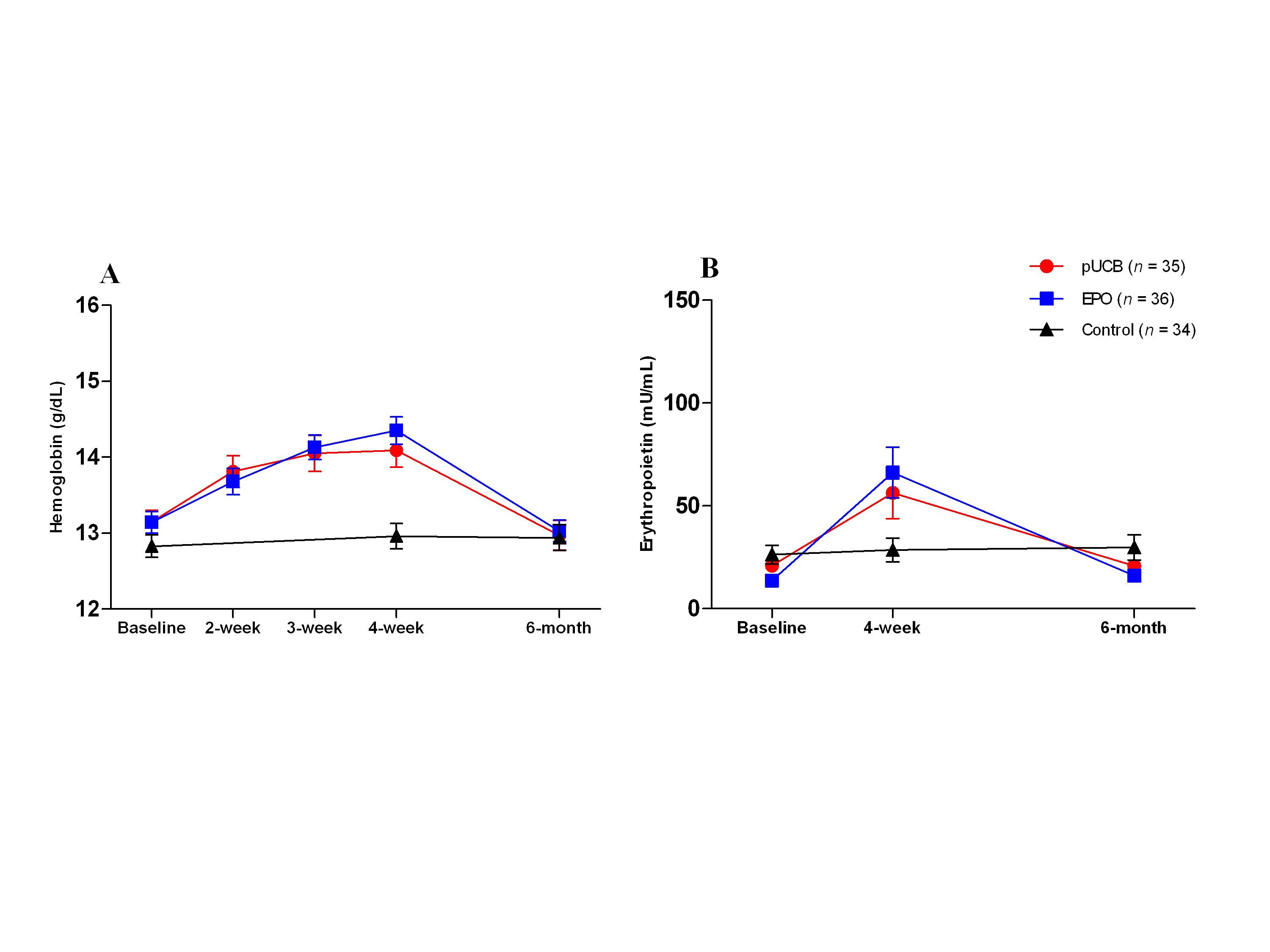

Supplement: Supplementary file 17 [file stem0031-0581-SD17.tif]

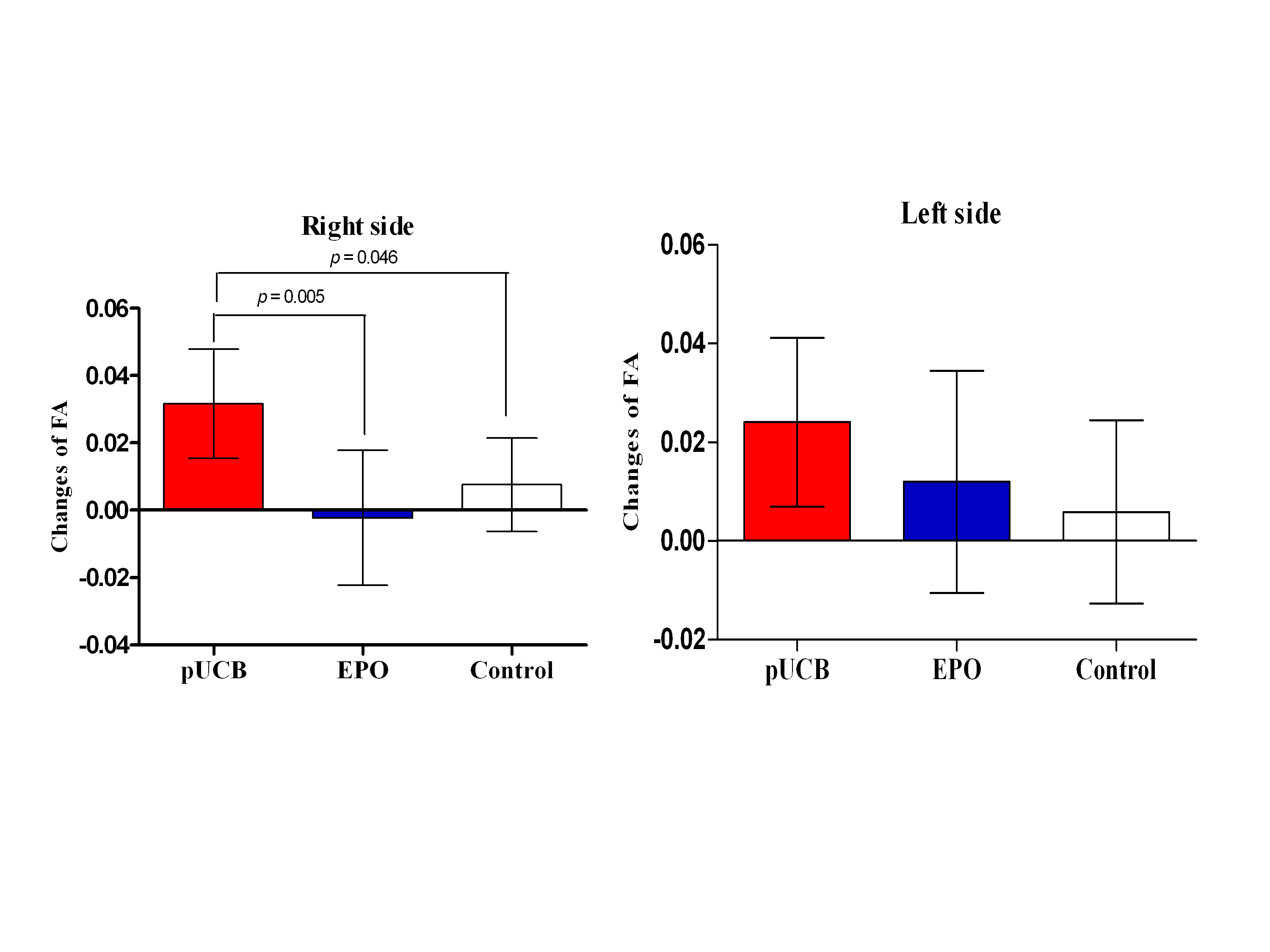

Supplement: Supplementary file 18 [file stem0031-0581-SD18.tif]
