# Supplementary material for: Umbilical Cord Blood Therapy Potentiated with Erythropoietin for Children with Cerebral Palsy: A Double-blind, Randomized, Placebo-Controlled Trial
Source: Stem Cells. 2012 Dec 24;31(3):581–91. doi: 10.1002/stem.1304 (PMC3744768; doi:10.1002/stem.1304)

pUCB group ($n$ = 31) versus EPO group ($n$ = 33)

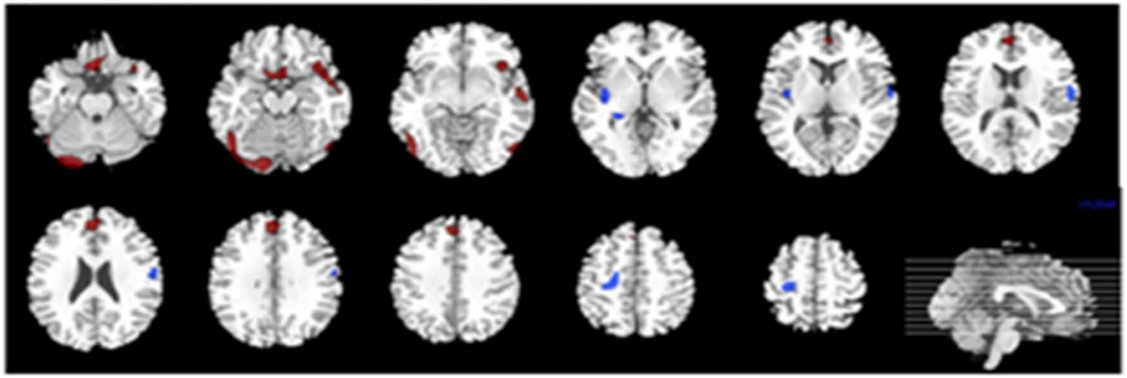

pUCB group ($n$ = 31) versus Control group ($n$ = 32)

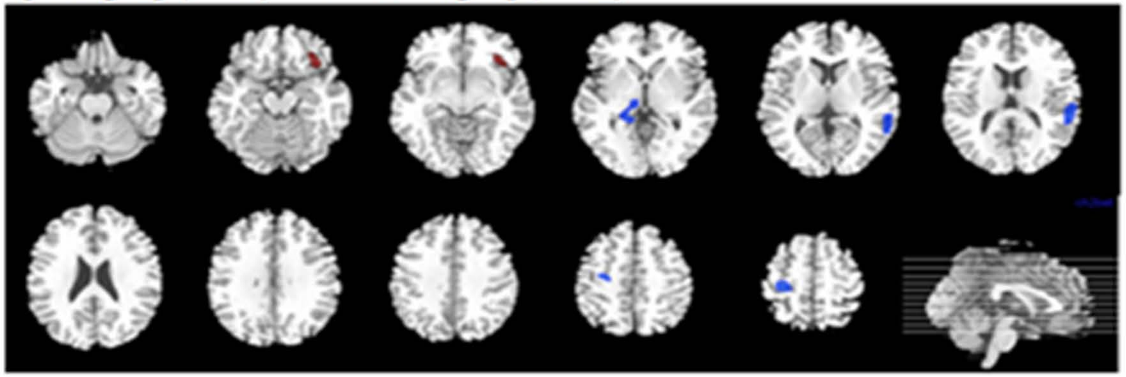

EPO group ($n$ = 33) versus Control group ($n$ = 32)

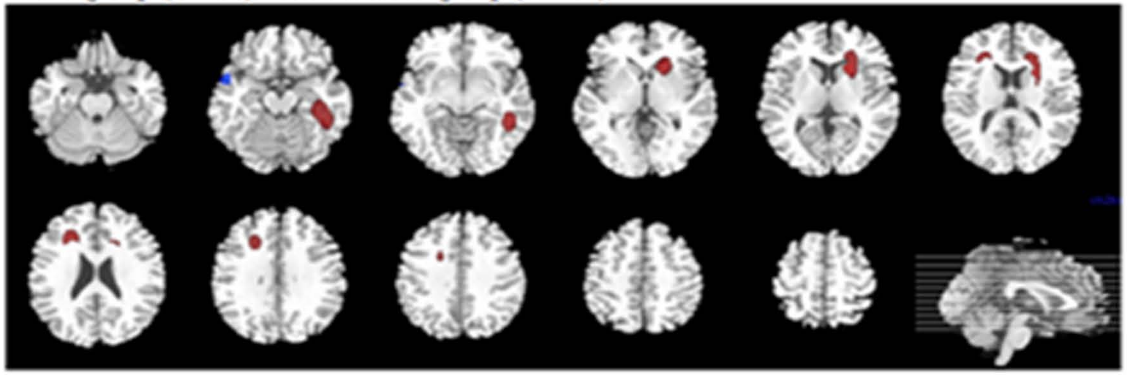

Supplement: Supplementary file 20 [file stem0031-0581-SD20.pdf]
